# Mapping the key residues of SufB and SufD essential for biosynthesis of iron-sulfur clusters

**DOI:** 10.1038/s41598-017-09846-2

**Published:** 2017-08-24

**Authors:** Eiki Yuda, Naoyuki Tanaka, Takashi Fujishiro, Nao Yokoyama, Kei Hirabayashi, Keiichi Fukuyama, Kei Wada, Yasuhiro Takahashi

**Affiliations:** 1 Department of Biochemistry and Molecular Biology, Graduate School of Science and Engineering, Saitama University, 255 Shimo-Okubo, Sakura-ku, Saitama 338-8570 Japan; 2 Department of Medical Sciences, University of Miyazaki, Miyazaki, 889-1692 Japan; 3 Department of Applied Chemistry, Graduate School of Engineering, Osaka University, Osaka, 565-0871 Japan; 4 Innovation Medical Research Institute, Present Address: University of Tsukuba, Ibaraki, 305-8577 Japan; 5 Department of Applied Biological Chemistry, Present Address: Graduate School of Agricultural and Life Sciences, The University of Tokyo, Tokyo, 113-8657 Japan

## Abstract

Biogenesis of iron-sulfur (Fe-S) clusters is an indispensable process in living cells. In *Escherichia coli*, the SUF biosynthetic system consists of six proteins among which SufB, SufC and SufD form the SufBCD complex, which serves as a scaffold for the assembly of nascent Fe-S cluster. Despite recent progress in biochemical and structural studies, little is known about the specific regions providing the scaffold. Here we present a systematic mutational analysis of SufB and SufD and map their critical residues in two distinct regions. One region is located on the N-terminal side of the β-helix core domain of SufB, where biochemical studies revealed that Cys254 of SufB (SufB^C254^) is essential for sulfur-transfer from SufE. Another functional region resides at an interface between SufB and SufD, where three residues (SufB^C405^, SufB^E434^, and SufD^H360^) appear to comprise the site for *de novo* cluster formation. Furthermore, we demonstrate a plausible tunnel in the β-helix core domain of SufB through which the sulfur species may be transferred from SufB^C254^ to SufB^C405^. In contrast, a canonical Fe-S cluster binding motif (CxxCxxxC) of SufB is dispensable. These findings provide new insights into the mechanism of Fe-S cluster assembly by the SufBCD complex.

## Introduction

Iron sulfur (Fe-S) proteins contribute to a number of cellular processes such as respiration, photosynthesis, nitrogen fixation and gene regulation^1–5^. Fe-S clusters come in three forms, [2Fe-2S], [4Fe-4S], and [3Fe-4S], and are assembled, in general, by coordination to cysteine residues of proteins but occasionally also to histidine, aspartate, serine or arginine residues^6, 7^. The biogenesis of Fe-S clusters in living cells is a highly complex and coordinated process. In bacteria, three distinct pathways for Fe-S cluster biosynthesis are the NIF system^8, 9^, the ISC system (also conserved in eukaryotic mitochondria)^10–13^, and the SUF system (also conserved in eukaryotic plastids)^14–17^. The three systems share some mechanistic aspects: the biosynthetic processes include mobilization of sulfur, formation of the nascent Fe-S cluster on a so-called scaffold protein, and delivery of the cluster to target proteins^18, 19^. *Escherichia coli* and closely related enterobacteria possess two pathways. The ISC system is encoded by the *iscRSUA-hscBA-fdx-iscX* operon and predominantly carries out the Fe-S cluster biosynthesis under normal conditions. The SUF system is encoded by the *sufABCDSE* operon whose expression is induced under oxidative stress and iron starved conditions^15, 20, 21^.

In the SUF machinery, SufS is a pyridoxal 5′-phosphate-containing cysteine desulfurase that mobilizes sulfur from the substrate L-cysteine, resulting in a persulfide (-SSH) species bound to an active cysteine residue SufS^C364^ ^22, 23^. SufE is a sulfur shuttle protein that interacts with SufS and accepts the persulfide species on the active site SufE^C51^ residue^24–28^. SufS alone shows basal cysteine desulfurase activity, but the activity is markedly enhanced by SufE. SufE also interacts with SufB and transfers persulfide for assembly of the Fe-S cluster^25, 29^. The SufS-SufE cysteine desulfurase activity is further enhanced by the addition of SufBCD, probably because the persulfide transfer to SufB allows SufE to accept another persulfide from SufS, which in turn allows SufS to initiate another round of catalysis. Polysulfurated SufB was demonstrated in the *in vitro* reaction consisting of L-cysteine, SufS, SufE and SufBCD; however, it is not known which residue of SufB accepts persulfide from SufE.

SufB forms a stable complex with SufC and SufD with a 1:2:1 stoichiometry, and acts as a scaffold for the *de novo* Fe-S cluster. The assertion that it performs a scaffold function is supported by the fact that the complex associated with an oxygen-labile Fe-S cluster has been purified^30^. In addition, the *in vitro* reconstituted Fe-S cluster in SufB has been found to readily transfer to several target proteins including SufA (an Fe-S carrier protein), Fdx, and aconitase^31–34^. The SufBCD complex also binds one equivalent of FADH_2_ per complex^30, 33^. It has been proposed that the reducing equivalent of FADH_2_ is utilized for the reduction of Fe^3+^ to Fe^2+^ or persulfide (S°) to sulfide (S^2−^). Upon oxidation, FAD is released from the SufBCD complex.

Recently, we have determined the crystal structure of the SufBCD complex^35^. SufB and SufD are homologous subunits (17% sequence identity) sharing a common domain organization: an N-terminal helical domain, a core domain consisting of a parallel β-helix, and a C-terminal helical domain. The core domains of SufB and SufD associate with each other forming an interface via two anti-parallel β-sheets between the subunits. SufC is a typical member of the nucleotide-binding subunit family involved in ATP-binding cassette (ABC) transporters^36, 37^. One SufC subunit is bound to the C-terminal helical domain of SufB and the other to the corresponding domain of SufD. Although the two bound SufC subunits are spatially separated in the crystalline state, biochemical studies in solution have shown that they form a transient dimer during the catalytic step of ATP binding and hydrolysis^35^. Furthermore, the SufC dimerization induces a conformational change of the SufB-SufD interface, leading to dissociation of the anti-parallel β-sheets connecting the two subunits. Mutational studies have underscored the importance of the ATPase activity of SufC for the *in vivo* biosynthesis of the Fe-S cluster^30, 35^, and have further revealed two critical residues, SufB^C405^ and SufD^H360^, both of which reside inside the β-helix core domain at the SufB-SufD interface^35, 38^. These findings led us to propose that the conformational change at the SufB-SufD interface is induced by the dimer formation of SufC, which leads to the exposure of the buried residues including SufB^C405^ and SufD^H360^, on which the nascent Fe-S cluster assembles. However, the precise roles of SufB^C405^ and SufD^H360^ remain to be elucidated. In addition, since the Fe-S cluster is coordinated in general by four, or at least by three residues, additional amino acids may be involved in cluster formation and need to be identified. Furthermore, *E. coli* SufB harbors another Fe-S cluster binding motif (CxxCxxxC) the function of which has yet to be clarified^29^.

Because of the crucial importance of SufB and SufD in Fe-S cluster biogenesis, we decided to explore the functional residues of these proteins using systematic alanine scanning mutagenesis in this study. Taking advantage of complementation assays using an *E. coli* mutant strain that can survive without Fe-S clusters^39^, we identified eight functional residues in SufB (including SufB^C405^) and one residue in SufD (SufD^H360^) that mapped to two distinct regions in the SufBCD structure. Six functional residues were found in the N-terminal region of the β-helix core domain of SufB (termed region A) and our biochemical study uncovered that one of them, SufB^C254^ is essential for the stimulation of SufS-SufE cysteine desulfurase activity and for the accumulation of sulfur moieties in the SufBCD complex, thus revealing the acceptor site for persulfide from SufE. The SufB-SufD interface provides another functional region (termed region B) in which SufB^E434^, a newly identified functional residue, appears to work in concert with nearby SufB^C405^ and SufD^H360^, serving as the site for *de novo* cluster formation. The two regions A and B are connected by a tunnel inside the β-helix core domain of SufB through which the sulfur moiety is likely transferred from SufB^C254^ to SufB^C405^. These findings suggest novel mechanistic implications for the Fe-S cluster assembly by the SufBCD complex.

## Results

### Identification of critical residues in SufB

For this study, the *E. coli sufABCDSE* operon was divided into *sufAB* and *sufCDSE*, and cloned into the compatible plasmids pBBR-*sufAB* and pRK-*sufCDSE*, respectively. The plasmids were introduced into the *E. coli* strain UT109 in which the chromosomal *suf* operon and the *isc* operon are deleted (Δ*sufABCDSE* Δ*iscUA-hscBA*). Generally, deletion of both operons in *E. coli* is lethal; however, UT109 harbors the plasmid pUMV22 Sp^r^ that carries three genes for the mevalonate (MVA) pathway cloned from *Streptomyces* sp. CL190, which allows UT109 to grow with an absolute dependence on MVA supplementation^39^. Upon introduction of the functional *sufAB* and *sufCDSE* genes (in this case from the plasmids pBBR-*sufAB* and pRK-*sufCDSE*, respectively) the cells become able to grow normally even in the absence of MVA.

**Figure 1:**
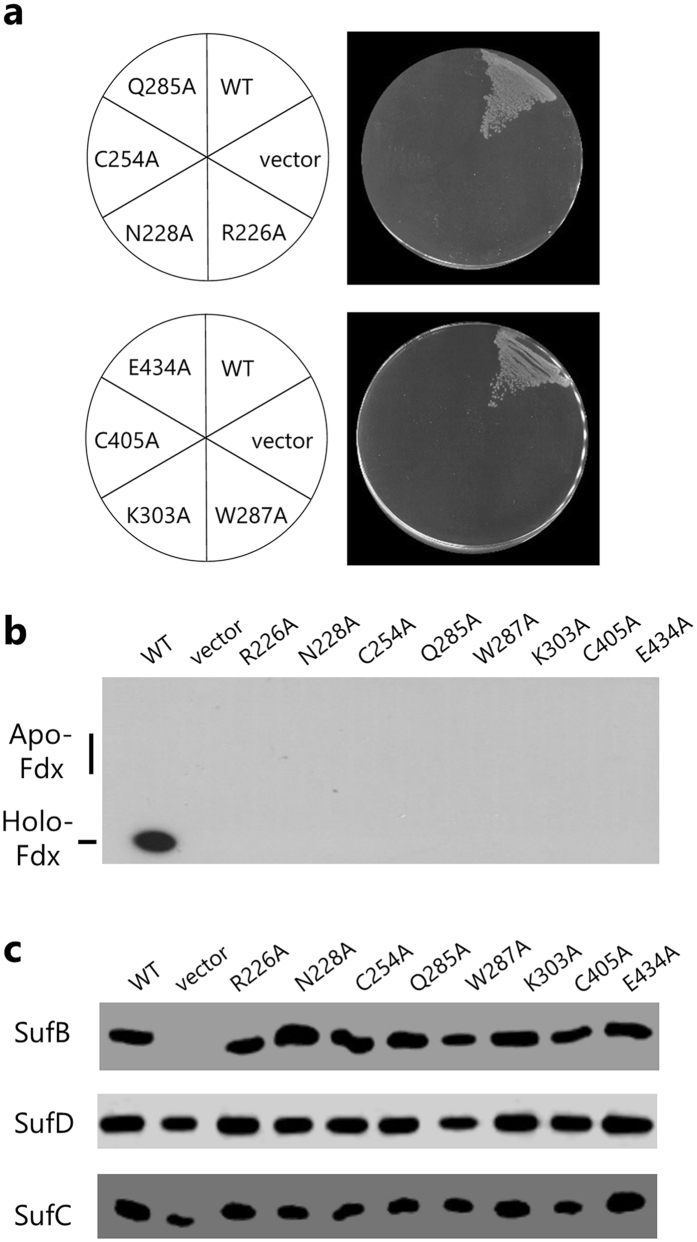
Effect of amino acid substitutions in SufB. (**a)** Growth phenotype of cells carrying mutations in *sufB*. The UT109 cells (Δ*isc* Δ*suf*) harboring pUMV22 Sp^r^ were sequentially transformed with the pRK-*sufCDSE* plasmid and the pBBR-*sufAB* plasmid carrying a point mutation in *sufB* (SufB^R226A^, SufB^N228A^, SufB^C254A^, SufB^Q285A^, SufB^W287A^, SufB^K303A^, SufB^C405A^, or SufB^E434A^). The cells were grown on LB plates (without MVA) at 37 °C for 24 h. **(b)** Effect of amino acid substitutions on the maturation of [2Fe-2S] Fdx. Cells were grown in liquid Superbroth supplemented with glucose and MVA. Bacterial extracts were normalized with respect to cell density and subjected to native PAGE to separate holo- and apo-Fdx. Fdx was detected by Western blot analysis using specific antibody. **(c)** Protein levels of SufB, SufD, and SufC determined by SDS-PAGE and Western analysis.

To identify functional residues of SufB, we generated a series of mutations to substitute several amino acids in SufB. Since the crystal structure of the SufBCD complex did not clearly indicate the potential binding site of the Fe-S cluster, Fe ion, or sulfur moiety, we first focused on the cysteine and histidine residues, the well-known ligands for the Fe-S cluster. We selected the nine cysteines (SufB^C167^, SufB^C217^, SufB^C254^, SufB^C307^, SufB^C332^, SufB^C405^, SufB^C414^, SufB^C448^, and SufB^C467^) and the five histidines (SufB^H176^, SufB^H265^, SufB^H363^, SufB^H417^, and SufB^H433^) that are more than 35% conserved among SufB orthologs based on phylogenetic alignment of 237 sequences. We also selected three cysteines (SufB^C96^, SufB^C99^, SufB^C103^) in the CxxCxxxC arrangement that resembles the canonical Fe-S cluster binding motif. In the crystal structure, this region is disordered and invisible^35^. Alanine scanning mutagenesis was performed using pBBR-*sufAB* as a template and the resultant plasmids were introduced into UT109 cells harboring pRK-*sufCDSE* and pUMV22 Sp^r^. The *in vivo* complementation assays revealed two critical cysteines, SufB^C254^ and SufB^C405^; the substitution of either of these residues (SufB^C254A^ or SufB^C405A^) prevented the UT109 cells from growing in the absence of MVA (Fig. 1a). By contrast, the other cysteines and histidines of SufB were revealed as nonessential since alanine substitution did not elicit any conspicuous effect (Supplementary Table S3). We also examined the deletion of eight residues from SufB^C96^ to SufB^C103^, and found, to our surprise, that the deletion of the entire CxxCxxxC motif (denoted SufB^∆96-103^ in Supplementary Table S3) had no effect on the complementation. The CxxCxxxC motif is thus dispensable for the *in vivo* function of SufB.

Next, we examined other amino acids that have S-, N- and/or O-containing side chains with the expectation that some of them would interact with Fe-S cluster or Fe ions. We selected 51 residues that are at least 75% conserved among the SufB orthologs. Six of these residues were found to be critical for SufB function, since the alanine substitutions SufB^R226A^, SufB^N228A^, SufB^Q285A^, SufB^W287A^, SufB^K303A^ or SufB^E434A^ abolished *in vivo* complementation (Fig. 1a). By contrast, alanine substitution of the remaining 45 amino acids did not prevent the cells from growing in the absence of MVA. Among them, the substitution of 25 amino acids did not cause noticeable defects, while the substitution of 20 amino acids caused a retarded growth phenotype, in which the complementation was partial (Supplementary Table S3). We also examined growth at higher temperature, and found that cells carrying the SufB^Y224A^, SufB^Q234A^, SufB^E236A^ or SufB^E252A^ substitution were not viable at 43 °C without MVA (Supplementary Fig. S1); in other words, these substitutions in SufB caused a temperature-sensitive growth phenotype.

The MVA-dependent growth phenotype clearly indicates the malfunction of the [4Fe-4S] enzymes IspG and IspH in the isoprenoid biosynthesis MEP pathway^39^. To examine the cluster formation in other Fe-S proteins, endogenous holo-Fdx, a [2Fe-2S] ferredoxin, was monitored by Western analysis, where we took advantage of the fact that holo- and apo-forms of Fdx are separated on native PAGE. As shown in Fig. 1b, holo-Fdx was found in the cells carrying a wild-type *sufB* gene. By contrast, no holo-form was detected in cells carrying amino acid substitutions in SufB that could not complement the MVA-dependent growth phenotype (SufB^R226A^, SufB^N228A^, SufB^C254A^, SufB^Q285A^, SufB^W287A^, SufB^K303A^, SufB^C405A^, or SufB^E434A^). Thus, these substitutions in SufB impaired the Fe-S cluster formation for proteins containing either [4Fe-4S] or [2Fe-2S] clusters. Apo-Fdx was not detected in any cells, which is likely due to the instability of the disordered state by lacking the cluster, probably leading to proteolysis. We also determined the protein level of SufB and its partner proteins SufC and SufD in cells cultivated in the presence of MVA. Almost no significant changes were observed in cells carrying the substitutions in SufB (Fig. 1c). These results underscore that the eight residues (SufB^R226^, SufB^N228^, SufB^C254^, SufB^Q285^, SufB^W287^, SufB^K303^, SufB^C405^, and SufB^E434^) are important for the function of SufB but not crucial for its stability.

We next examined whether the critical residues of SufB could be functionally replaced with any other amino acids. To this end, the corresponding codons that were substituted by the alanine codon in the plasmid pBBR-*sufAB* were further replaced randomly with the NNK codon, where K = G or T. The mutated plasmids were introduced into UT109 cells harboring pUMV22 Sp^r^ and pRK-*sufCDSE*, and recovered from cells that could grow in the absence of MVA. Sequencing of these plasmids revealed that SufB^R226^ could be functionally replaced with valine, isoleucine, and leucine (Supplementary Table S4). Likewise, we observed functional replacement of SufB^N228^ with cysteine, histidine, and glutamine, of SufB^Q285^ with glycine, asparagine, and arginine, of SufB^W287^ with tyrosine, methionine, and phenylalanine, of SufB^K303^ with glutamate and arginine, and of SufB^E434^ with aspartate. It should be noted that these substitutions did not fully restore SufB function, as the cells carrying these suppressor mutations exhibited retarded growth phenotype (Supplementary Table S4). By contrast, sequencing of 30 and 27 plasmids recovered from the SufB^C254^ and SufB^C405^ mutagenesis, respectively, resulted in regeneration of the original cysteine codon alone. Thus, SufB^C254^ and SufB^C405^ are the essential residues for SufB function, which cannot be substituted by any other of the 19 amino acids.

### Mutational studies of SufD and the SufB-SufD interface

We had previously carried out mutational studies of SufD and reported that one residue, SufD^H360^, was essential for its function^38^. However, we had used a temperature-sensitive complementing plasmid in the experiments, and could thus not determine whether the SufD^H360^ substitution caused functional deficits or thermal instability. In addition, the crystal structure of the SufBCD complex indicates that SufD^H360^ resides at the SufB-SufD interface facing SufB^E434^, one of the functional residues of SufB mentioned above. Therefore, we re-examined the effect of the SufD^H360A^ substitution, and further screened other functional residues of SufD by complementation analysis of UT109 using the plasmids pBBR-*sufD* and pRK-*sufABC-SE*. It turned out that the SufD^H360A^ substitution did not complement the MVA-dependent growth phenotype at 37 °C, nor the maturation failure of [2Fe-2S] Fdx (Supplementary Fig. S2). The protein level of the SufD variant carrying the SufD^H360A^ substitution was slightly lower, but not all that much lower, than that of the normal SufD (Supplementary Fig. S2c). These results confirm the essential role of SufD^H360^ for the function of SufD. By contrast, screening for other functional residues of SufD was unsuccessful. We selected amino acids that have S-, N- and/or O-containing side chains and are more than 60% conserved among the SufD orthologs. Alanine-substitution of these residues (SufD^D283A^, SufD^H290A^, SufD^K302A^, SufD^D344A^, SufD^E350A^, SufD^D354A^, SufD^D355A^, or SufD^C358A^) did not cause any noticeable defects in the growth of the complemented cells (Supplementary Table S3 and Fig. S2a).

**Figure 2:**
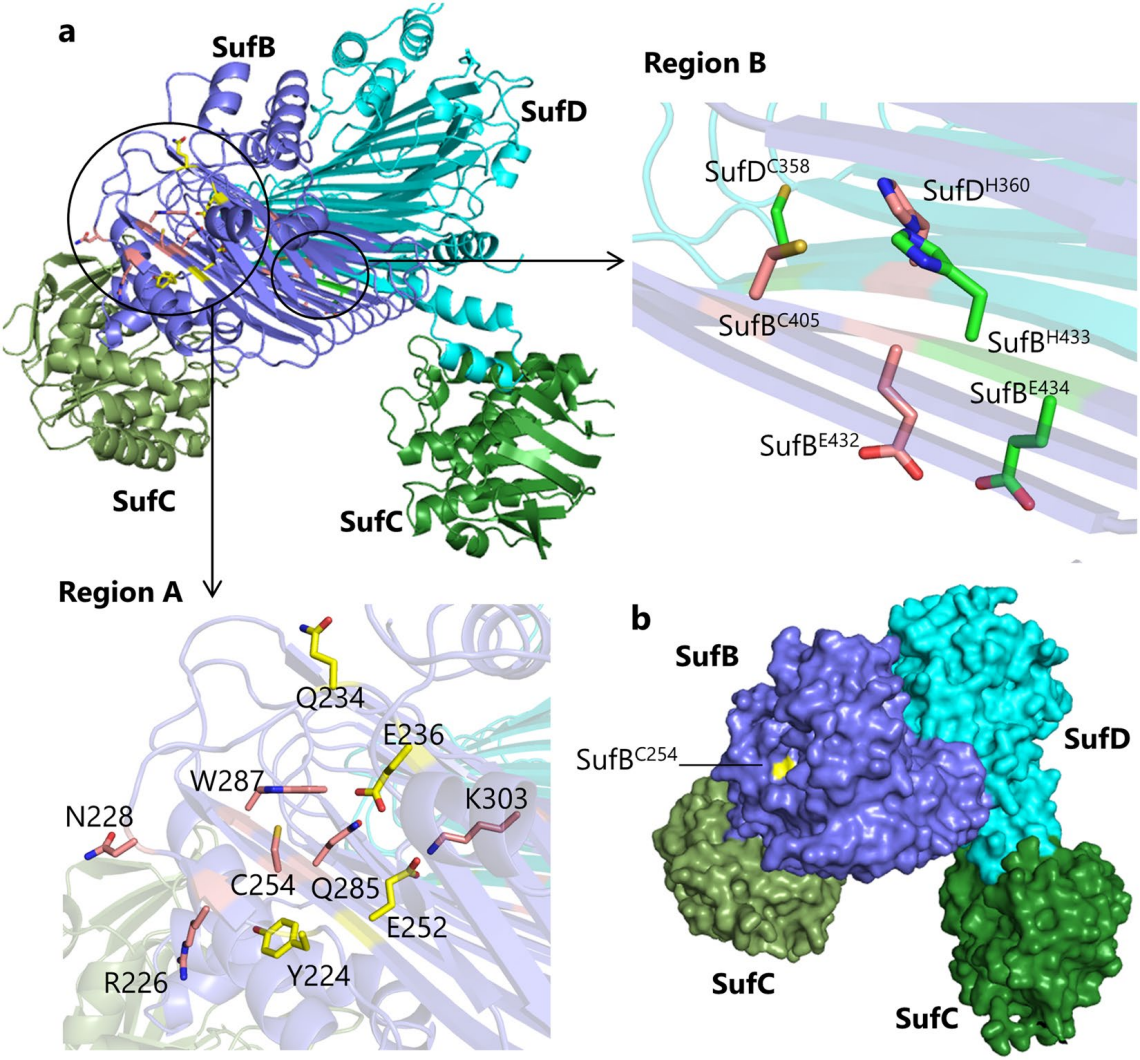
Functional residues of SufB and SufD in the SufBCD complex. (**a)** Distinct distributions of the functional residues depicted in the crystal structure of the SufBCD complex (PDB code: 5AWF). Region A is located at the N-terminal part of the β-helix core domain of SufB and contains six critical residues of SufB (SufB^R226^, SufB^N228^, SufB^C254^, SufB^Q285^, SufB^W287^, and SufB^K303^; depicted in pink), among which SufB^C254^ was found to be essential for the sulfur-transfer from SufE. Temperature-sensitive mutations are also located in this region (SufB^Y224^, SufB^Q234^, SufB^E236^, and SufB^E252^; yellow). Region B is located at the interface between SufB and SufD and contains three critical residues (SufB^C405^, SufB^E434^, and SufD^H360^; pink). In addition, SufB^E432^, SufB^H433^ and SufD^C358^ (green) residing in region B appear to share a redundant role. **(b)** Surface representation of the SufBCD complex. The SufB^C254^ residue is located in a pit on the N-terminal side of the β-helix core domain of SufB. The side chain is shown in yellow.

**Figure 3:**
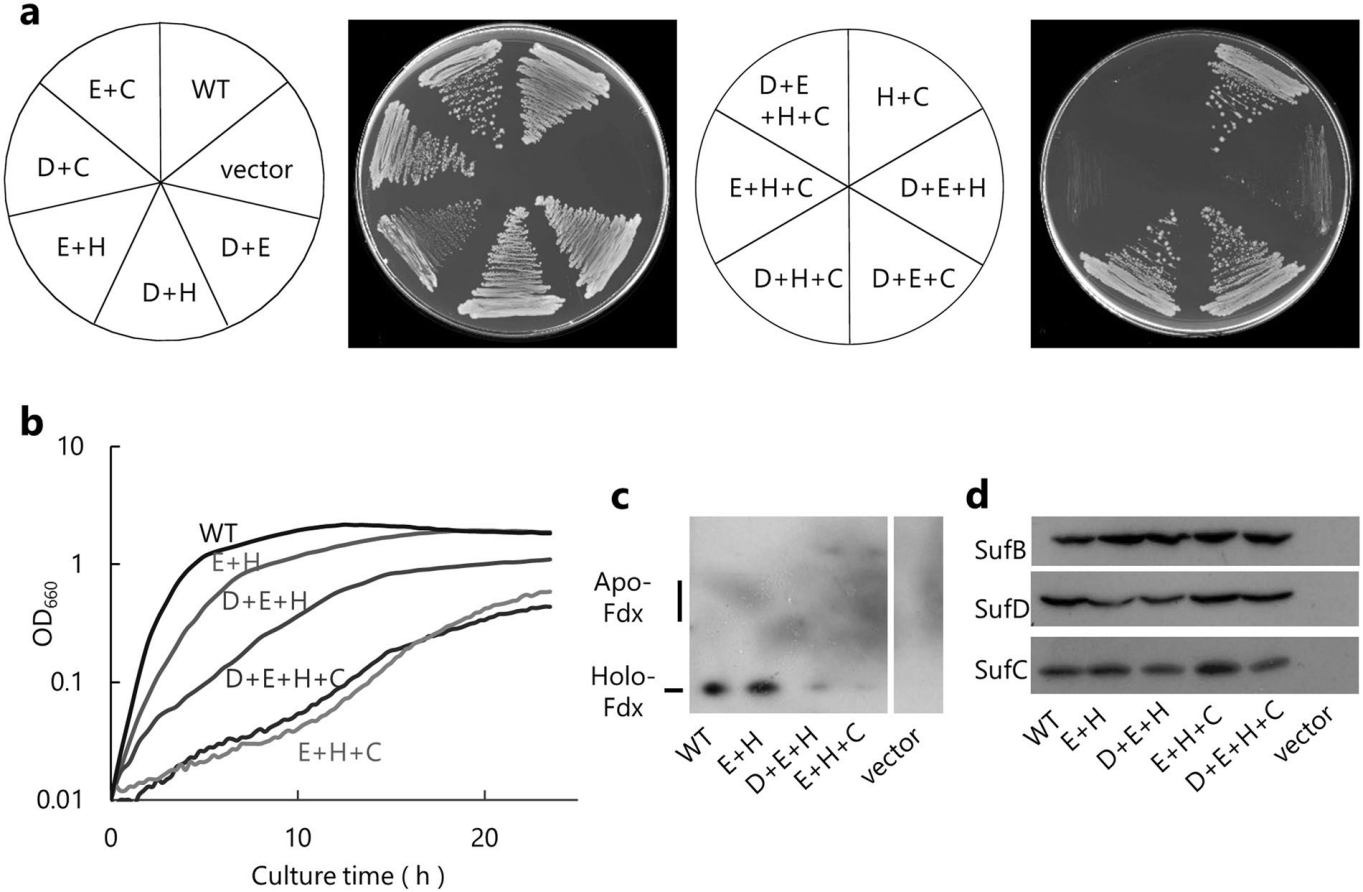
Effect of multiple substitutions at the SufB-SufD interface. Four conserved residues were combinatorially substituted (D, SufB^D406A^; E, SufB^E432A^; H, SufB^H433A^; and C, SufD^C358S^) using the plasmids pBBR-*sufAB* and pRK-*sufCDSE*. The plasmids were introduced into UT109 cells harboring pUMV22 Sp^r^. **(a)** The cells were grown on LB plates (without MVA) at 37 °C for 24 h. **(b)** The cells were grown in liquid LB (without MVA) at 37 °C and monitored by optical density at 660 nm (OD_660_). **(c)** [2Fe-2S] holo-Fdx, and **(d)** protein levels of SufB, SufD and SufC were determined as described in the legend to Fig. 1.

Next, we focused on the interface between SufB and SufD, where three functional residues SufB^C405^, SufB^E434^ and SufD^H360^ reside close to each other (Fig. 2). These are the potential ligands for the Fe-S cluster, and furthermore, our previous biochemical studies demonstrated a conformational change in this region that is driven by the dimerization of SufC in the presence of ATP^35^. To further identify functional residues in this region, we introduced multiple amino acid substitutions using the plasmids pBBR-*sufAB* and pRK-*sufCDSE*. Although the single substitution SufB^D406A^, SufB^E432A^, SufB^H433A^, or SufD^C358S^ had no significant effect on the growth of complemented cells as described above, combination of these substitutions elicited slow-growth phenotypes in the absence of MVA. Among them, a severe defect was observed for the combination SufB^D406A^, SufB^E432A^ and SufB^H433A^, and an even more severe defect when SufB^E432A^, SufB^H433A^ and SufD^C358S^ were combined, while the combination of all four substitutions did not work additively (Fig. 3a and b). Formation of [2Fe-2S] cluster of Fdx was also impaired in cells carrying the triple substitutions, although a faint band of holo-Fdx was detected (Fig. 3c). The protein levels of SufB and SufD carrying the triple substitutions were almost comparable to those of the normal proteins (Fig. 3d). These results suggest that SufB^E432^, SufB^H433^, and SufD^C358^ share a redundant role that is critical but not essential for the biosynthesis of Fe-S clusters.

### Role of SufB^C254^ in sulfur trafficking from SufE

To further examine the roles of the functional residues of SufB, the SufBCD complexes carrying the loss-of-function substitutions were purified and biochemically characterized. The variant complexes were eluted from the gel filtration column at around a position corresponding to a molecular mass of 160 kDa (Supplementary Fig. S3a). SDS-PAGE of the purified sample confirmed that the variant complexes were composed of SufB, SufC and SufD with the same 1:2:1 stoichiometry that is observed for the wild-type complex (Supplementary Fig. S3b). We conclude that the loss-of-function substitutions in SufB did not impair the assembly of the complex.

**Figure 4:**
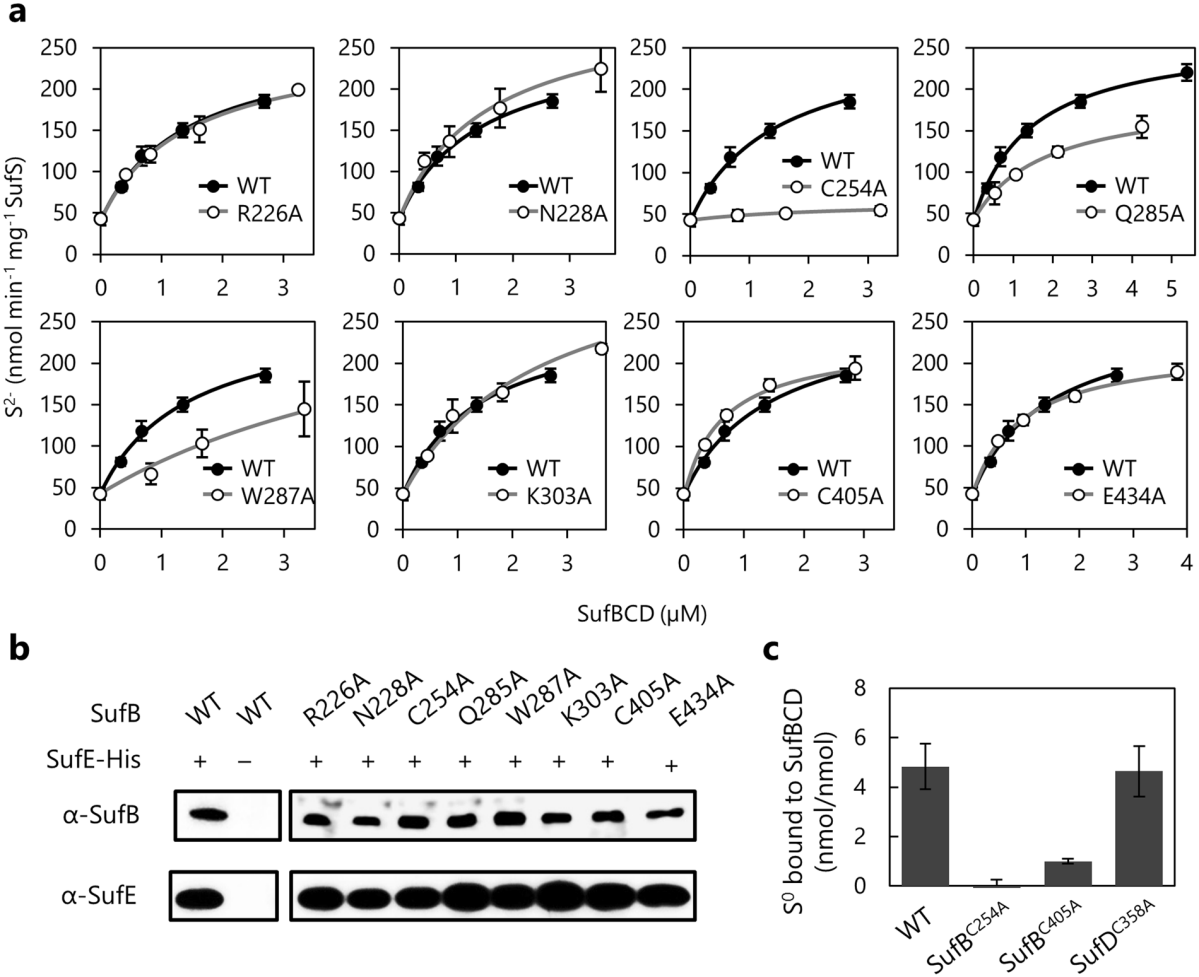
Effect of amino acid substitutions on the interaction between SufSE and SufBCD. (**a)** Stimulation of SufS cysteine desulfurase activity. The reactions contained 0.5 µM SufS, 2.0 µM SufE, 2 mM L-cysteine, 2 mM DTT, and various concentrations of SufBCD. After incubation for 10 min at 30 °C, cysteine desulfurase activity was determined by quantifying sulfide via formation of methylene blue. Values are the mean ± SD of at least three measurements. **(b)** Pull-down assays between SufB and SufE-(His)_6_. The plasmids pBBR*-sufAB* carrying mutations in *sufB* and pRK-*sufCDSE*-His were introduced into YT2512 (∆*sufABCDSE*) cells. Bacterial extracts were prepared from cells cultivated in LB and normalized with respect to cell density. The SufE-(His)_6_ proteins were purified by Ni-resin and co-purified SufB was examined by Western blot analysis. **(c)** Persulfuration of the SufBCD complex. The sulfur-transfer reaction contained 10 µM SufBCD, 0.5 µM SufS, 0.5 µM SufE, and 2 mM L-cysteine. After incubation at 30 °C for 3 h, the SufBCD complex was separated from other components by ultrafiltration with 100 kDa cutoff Amicon Ultra membrane (Millipore). The bound S^0^ was reduced by DTT and the resultant sulfide was determined by methylene blue colorimetric assay. Values are the mean ± SD of at least three measurements.

In our *in vitro* assay of cysteine desulfurase, SufE enhanced SufS activity up to 11-fold, to which the addition of the wild-type SufBCD complex added a further up to 4.3-fold increase (data not shown). These results are in good agreement with previous experiments reporting that SufE enhanced SufS activity up to 8-fold and further addition of SufBCD enhanced it up to 4-fold again^25^. The variant SufBCD complex carrying the SufB^R226A^, SufB^N228A^, SufB^K303A^, SufB^C405A^ or SufB^E434A^ substitution enhanced the SufS cysteine desulfurase activity as much as the wild-type complex (Fig. 4a). In contrast to this, addition of the variant complex carrying a SufB^Q285A^ or SufB^W287A^ substitution resulted in an only modest increase in the activity. Notably, no enhancement was observed for the variant complex carrying SufB^C254A^ (Fig. 4a).

To examine physical interaction between SufE and the variant SufBCD complexes, SufE-(His)_6_ was expressed with other SUF members, including the SufB variants in *E. coli* mutant cells where the chromosomal *sufABCDSE* operon was deleted. Pull-down assays demonstrated that the variant forms including SufB^Q285A^, SufB^W287A^ and SufB^C254A^ were all co-purified with SufE-(His)_6_, suggesting that these substitutions did not disrupt the physical contact between SufB and SufE (Fig. 4b).

Next, we determined S^0^ that was transferred from SufSE to the SufBCD complex. In the experiments, purified SufS, SufE, and the SufBCD complex were incubated with the substrate L-cysteine in the absence of the reducing agent DTT, allowing persulfide accumulation in the SufBCD complex. The persulfurated SufBCD complex was isolated by ultrafiltration with a 100 kDa cut-off filter from which SufS, SufE and the substrate L-cysteine were washed away. Then the bound S^0^ was reduced to S^2−^ and measured by methylene blue formation. The wild-type SufBCD accumulated 4.8 ± 0.9 bound S^0^ atoms per complex. The variant complex carrying SufD^C358A^ also accumulated 4.6 ± 1.0 S^0^ atoms per complex, whereas the complex carrying SufB^C405A^ had decidedly less S^0^ with only 1.0 ± 0.1 atoms per complex. Most remarkably, no S^0^ was detected in the variant SufBCD complex carrying the SufB^C254A^ (Fig. 4c). Taken together, these results indicate that the SufB^C254A^ substitution abolished the sulfur transfer from SufE.

## Discussion

In this study, we identified eight functional residues of SufB, which form two groups, clearly distributed into two separate regions of SufB; one is in the N-terminal area of the β-helix core domain (termed region A), and the other in the C-terminal area of the same domain (termed region B) (Fig. 2a). Region A contains six functional residues (SufB^R226^, SufB^N228^, SufB^C254^, SufB^Q285^, SufB^W287^, and SufB^K303^), one of which, SufB^C254^ was found to be essential in that it could not be functionally substituted with any other amino acid. We conclude that SufB^C254^ serves as the acceptor site for persulfide transferred from SufE based on the following evidence. The SufB^C254A^ substitution abolished the activation of SufS cysteine desulfurase in the *in vitro* assay system composed of SufS, SufE, and the SufBCD complex (Fig. 4a). Previous studies by Outten’s group have demonstrated that S^0^ is transferred from SufS to SufE, and then to SufB in the form of protein-bound cysteinyl persulfide (-SSH) in a manner that is shielded from solvent (therefore from reduction by DTT), via a specific protein-protein interaction^25, 28, 29^. Our results were in good agreement with these experiments and further demonstrated that the SufBCD complex accumulated nearly five sulfur atoms per complex when DTT was omitted from the reaction. The sulfur moiety will presumably be utilized for the assembly of Fe-S clusters when adequate Fe ions are available. The present study demonstrated that the SufB^C254A^ substitution even abolished the accumulation of S^0^ in the SufBCD complex in the reaction without DTT (Fig. 4c). We also observed that the SufB^C254A^ substitution hardly affected the expression level of SufB, SufC and SufD proteins (Fig. 1c), the assembly of the SufBCD complex (Supplementary Fig. S3), or the physical interaction with SufE (Fig. 4b). Thus, we concluded that the SufB^C254A^ substitution directly blocked the sulfur trafficking from SufE to SufB. Based on the crystal structure, the side chain of SufB^C254^ resides in the β-helix, but its Sγ atom is exposed to solvent and accessible from the N-terminal side of the core domain (Fig. 2b). The SufB^Q285^ and SufB^W287^ residues are positioned in close vicinity to SufB^C254^ (Fig. 2a), which may explain why their substitutions elicited modest but significant defects in the activation of cysteine desulfurase (Fig. 4a), supporting the notion that SufB^C254^ and its surroundings are responsible for the specific protein-protein interaction with SufE for trans-persulfuration.

Although one may speculate that region A could also serve as a scaffold for the *de novo* assembly of the Fe-S cluster, this seems unlikely. In general, an Fe-S cluster is coordinated by three or four ligand residues in which cysteine is predominantly utilized, while histidine, aspartate, arginine, threonine or serine occurs only occasionally^6^. Among the functional residues in region A, SufB^C254^ and SufB^R226^ are the potential ligand residues; however, SufB^R226^ could be functionally substituted with valine, isoleucine, or leucine (Supplementary Table S4), making it less likely that SufB^R226^ is involved in the coordination of the Fe-S cluster. Another hypothesis is that the functional residues in region A are involved in the interaction with FADH_2_. It has been demonstrated that anaerobically prepared SufBCD contains one FADH_2_ per complex^30, 33^. The exact role of FADH_2_ is not known, but reduction of Fe^3+^ to Fe^2+^ or S^0^ to S^2−^ has been proposed. A putative FADH_2_ binding motif has been suggested on the basis of sequence comparison with the *p*-cresol methylhydroxylase family^33, 40^, which has an R(x)_6_ExxY(x)_5_G(x)_8_Y motif that overlaps with region A of SufB (Supplementary Fig. S4). However, the overall folding of SufB and the local conformation of the motif is completely different from what one finds in the *p*-cresol methylhydroxylase family. Furthermore, substitution of the residues in this motif, SufB^R237A^ or SufB^E244A^, did not elicit any conspicuous consequences (Supplementary Table S3). Similarly, the substitution SufB^R451A^, of another residue proposed to interact with FADH_2_^33^, had no effect. Hence, the assignment of the FADH_2_ binding site requires further studies.

We found three critical residues (SufB^C405^, SufB^E434^ and SufD^H360^) in region B at the interface between SufB and SufD, which consists primarily of hydrogen-bonds in the two anti-parallel β-sheets (Fig. 2a). SufB^C405^ and SufD^H360^ were found to be essential as they could not be functionally substituted with any other amino acids, while SufB^E434^ could be substituted only with aspartate (Supplementary Table S4). In the crystallographic analysis of the SufBCD complex, we had previously identified two Hg^2+^ ions in this region during the course of phase determination using heavy atoms: one Hg^2+^ ion bound to SufB^C405^ and the other to SufD^C358^, and consequently we proposed that SufB^C405^ and SufD^C358^, together with nearby SufD^H360^, may serve as the assembly site for the nascent Fe-S cluster^35^. The present findings underscore the essential role of SufB^C405^ and SufD^H360^, and in addition, a third critical residue, SufB^E434^, was also identified. Although SufD^C358^ appears to be less critical, the triple substitution (SufB^E432A^, SufB^H433A^ and SufD^C358S^) resulted in a severe functional defect (Fig. 3). Most likely, SufB^C405^, SufB^E434^ and SufD^H360^ serve as the fixed ligands for the *de novo* Fe-S cluster assembly, whereas the fourth ligand seems to be flexible and exchangeable among SufB^E432^, SufB^H433^ and SufD^C358^. It is noteworthy that the three cysteine residues (SufB^C96^, SufB^C99^, SufB^C103^) in the CxxCxxxC motif of SufB were found dispensable in the *in vivo* complementation assay (Supplementary Table S3), suggesting that they do not serve as the Fe-S cluster assembly site. Supporting this view is the observation that the CxxCxxxC motif is not highly conserved among SufB sequences (Supplementary Fig. S4)^29^. It should also be noted that the [4Fe-4S] cluster was reconstituted anaerobically *in vitro* in the SufBCD complex and characterized by Mössbauer spectroscopy to have cysteinyl ligands^33^. However, anaerobically isolated SufBCD complex from *E. coli* was demonstrated to possess Fe-S cluster with distinct spectroscopic features from that of [4Fe-4S] cluster reconstituted *in vitro*. The cluster in the as-isolated complex was preliminarily assigned as a linear [3Fe-4S] cluster, but has yet to be characterized^30^.

**Figure 5:**
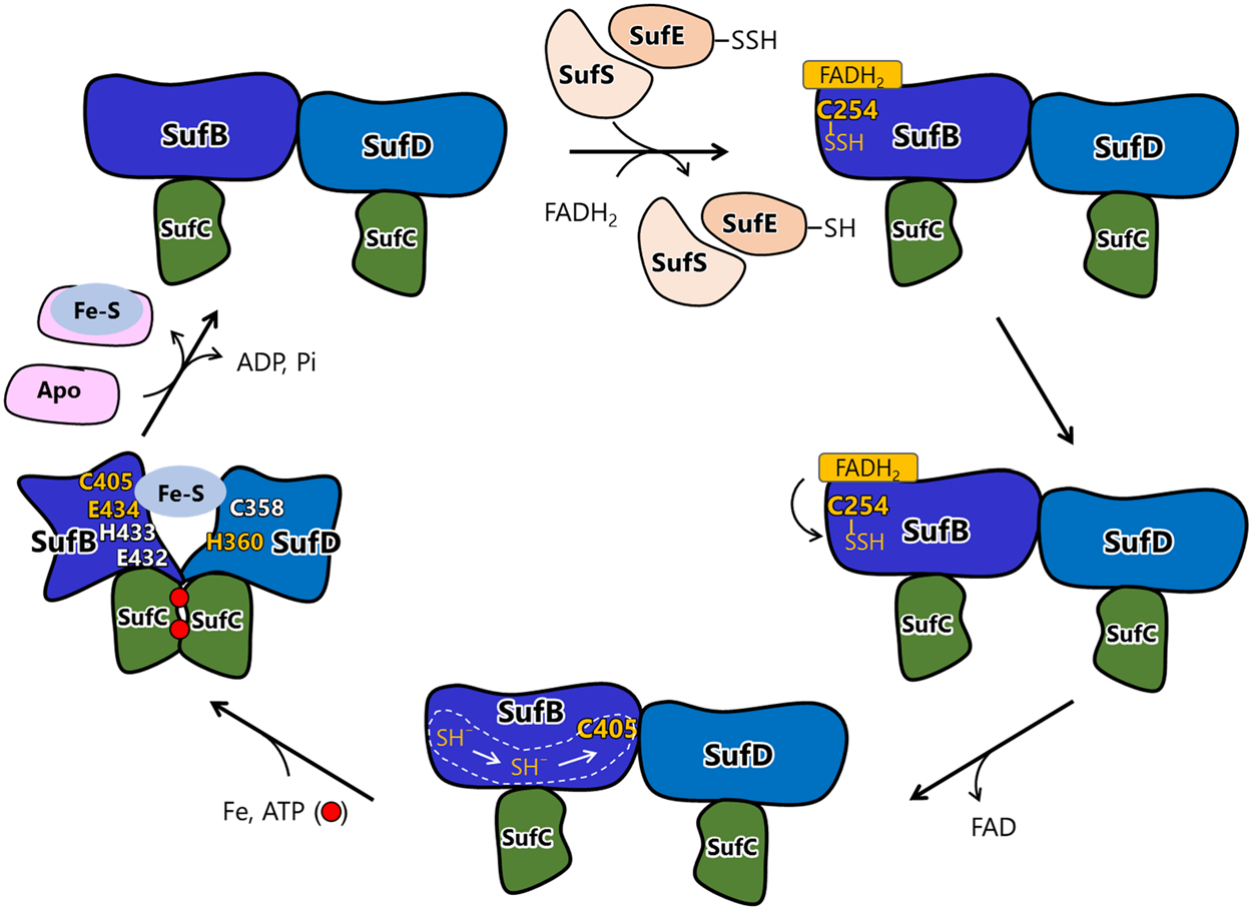
Model for the role of the SufBCD complex during Fe-S cluster biosynthesis. Sulfur (S^0^) is extracted from the substrate L-cysteine by the action of SufS and delivered via SufE to SufB^C254^ of the SufBCD complex in the form of persulfide (-SSH). The bound S^0^ is reduced to S^2−^, presumably by FADH_2_, released from SufB^C254^, and migrates through the hydrophilic tunnel that traverses the β-helix core domain of SufB from SufB^C254^ to SufB^C405^. Dimerization of SufC occurs upon binding to ATP, which induces a large conformational change at the SufB-SufD interface, where the Fe-S cluster is assembled using SufB^C405^, SufB^E434^ and SufD^H360^ as essential ligands in combination with a fourth redundant ligand that is provided by one of the three residues, SufB^E432^, SufB^H433^, or SufD^C358^.

The geometry of the potential ligands in region B does not make it feasible for them to bind an Fe-S cluster: the side chains SufB^C405^ and SufD^H360^ are buried inside the β-helix, whereas SufB^E434^ protrudes outside (Fig. 2a). However, we have recently demonstrated that the two SufC subunits, which are spatially separated in the crystal structure of the SufBCD complex, form a head-to-tail dimer in solution upon ATP binding^35^. The dynamic motion is transmitted to the SufB-SufD interface where a large conformational change occurs. Specifically, SufB^C405^ is exposed at the surface, which was detected by fluorescent thiol reagent^35^. Such a conformational change would rearrange the ligand residues, allowing them to assemble the nascent Fe-S cluster (Fig. 5). Further studies are required to elucidate the dynamic motion of the SufBCD complex and confirm this scenario.

**Figure 6:**
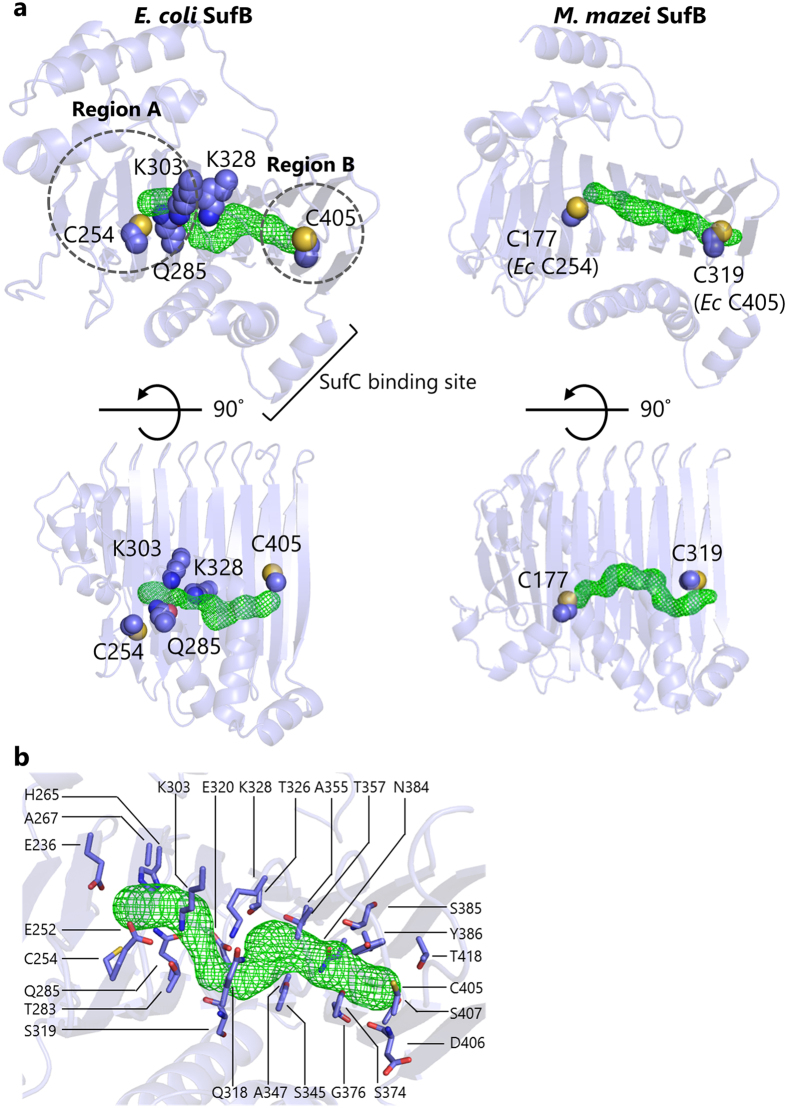
Putative sulfur tunnel ranging through the β-helix core domain of SufB. (**a**) *Left panel, E. coli* SufB. *Right panel, M. mazei* SufB (PDB code: 4DN7). The tunnels were detected using CAVER 3.01^42–44^ with a minimum probe radius of 1.04 Å. The tunnels are shown as green mesh. Critical amino acid residues are indicated in sphere models. **(b)** Close-up view of the hydrophilic tunnel in *E. coli* SufB. The orientation is the same as in (**a**) upper left panel. Amino acid residues surrounding the tunnel are depicted in stick models and labeled.

Another important aspect is the intramolecular sulfur-transfer from SufB^C254^ to region B. We observed that SufB^C405A^ substitution resulted in reduced S^0^ accumulation in the SufBCD complex to less than one fourth (Fig. 4c), suggesting that SufB^C405^ is involved in the sulfur transfer, probably serving as the acceptor site of the sulfur moiety transferred from SufB^C254^. However, SufB^C405^ is >25 Å away from SufB^C254^. Although SufB^C307^ and SufB^C332^ are located in the middle area between SufB^C254^ and SufB^C405^ of the β-helix core domain, the SufB^C307A^ and SufB^C332A^ double substitution did not have any phenotypic consequences (Supplementary Table S3), suggesting that these residues are not involved in the sulfur transfer. Instead, inspection of the crystal structure of the SufBCD complex led us to identify an internal tunnel ranging through the β-helix core domain of SufB just between SufB^C254^ and SufB^C405^ (Fig. 6a) The tunnel is lined primarily with hydrophilic side chains (Fig. 6b and Supplementary Table S5). Importantly, alanine-substitution of several residues surrounding the tunnel resulted in severe functional defects of SufB in our *in vivo* complementation assays; the SufB^Q285A^ or SufB^K303A^ substitution led to functional loss and other substitutions (SufB^E236A^, SufB^E252A^, SufB^H265A^, SufB^T283A^, SufB^T326A^, or SufB^K328A^) caused partial but significant deficits. Among these residues, SufB^K328^ is positioned at the bottleneck and may modulate the gate opening. No such tunnel was found in the core domain of SufD. By contrast, we also found a similar tunnel in the crystal structure of the SufB homodimer from *Methanosarcina mazei* Go1 (Fig. 6a). It should be noted that the tunnel in *M. mazei* SufB also ranges from C177 (corresponding to *E. coli* SufB^C254^) to C319 (corresponding to *E. coli* SufB^C405^) despite the very low level of sequence conservation between the two residues (Supplementary Fig. S4). Taken together, our findings suggest that the evolutionary conserved tunnel may allow intramolecular sulfur transfer through the β-helix core domain of SufB (Fig. 5). Future studies are necessary to clarify the specific role of the tunnel in the SufBCD complex.

## Methods

### Bacterial strains and cell growth

The *E. coli* K-12 strain MG1655 and its derivatives used in this study are listed in Supplementary Table S1. Luria-Bertani broth (LB) was used as the standard medium. The UT109 strain (∆*iscUA-hscBA* ∆*sufABCDSE*)^9^ harboring the pUMV22 Sp^r^ plasmid^39^ was cultivated on LB agar plates supplemented with 0.4% glucose and 200 µM (±) mevalonolactone (Sigma-Aldrich). The mutant was cultivated in liquid Superbroth (3.2% bacto tryptone, 2% yeast extract, 0.5% NaCl) supplemented with glucose and MVA. When required, ampicillin (Ap), tetracycline (Tc), and spectinomycin (Sp) were added at concentrations of 50, 10, and 40 µg/ml, respectively. Bacterial growth was monitored as described elsewhere^39^.

### Plasmid construction

The plasmids and primers used in this study are listed in Supplementary Tables S1 and S2, respectively. The *sufCDSE* fragment was amplified by PCR using the primers SufC-FSc2 and SufE-RNh. The fragment was digested with NheI and SacI and ligated into the corresponding sites of pRKNMC^11^ to generate pRK-*sufCDSE*. The *sufAB* fragment was amplified by PCR using the plasmid pRKSUF017^14^ as a template and the primers M13Rev and SufB-RSc. The PCR fragment was digested with XbaI and SacI and ligated into the corresponding sites of pBBR1MCS-4^41^ to generate the pBBR-*sufAB* plasmid. Site-directed mutagenesis of SufB was performed by inverse PCR using the pBBR-*sufAB* plasmid as a template and the primers listed in Supplementary Table S2. Site-directed mutations were also introduced into the pGSO164 plasmid^25^ for purification of the variant SufBCD complexes. Mutagenesis of SufD was performed using the pBBR-*sufD* plasmid^38^ as a template. For pull-down assays, the (His)_6_-tag sequence was fused to the C-terminus of SufE by inverse PCR using the pRK-*sufCDSE* plasmid as a template and the primers SufE-Cter-R and pRK-His6-F, generating the plasmid pRK-*sufCDSE*-His. For purification of the SufE protein (without His-tag sequence), the coding region of *sufE* was amplified by PCR using the primers EcSufE-F and EcSufE-R, and cloned into the pCR2.1-TOPO vector (Invitrogen) by the TA cloning method. Then the NdeI-BamHI fragment was excised and cloned into the pET-21(a) vector (Novagen) to construct pET21a-*sufE*. The nucleotide sequences of all the cloned fragments in the plasmids were confirmed by DNA sequencing.

### *In vivo* complementation assay

The complementation test was carried out essentially as previously described^39^. *E. coli* mutant strain UT109 (∆*iscUA-hscBA* ∆*sufABCDSE*) harboring the pUMV22 Sp^r^ plasmid was sequentially transformed by electroporation with the plasmids pRK-*sufCDSE* and pBBR-*sufAB* (carrying mutations) or pRK-*sufABC-SE* and pBBR-*sufD* (carrying mutations). The transformants were cultivated on LB agar in the presence and absence of MVA and glucose. For protein analysis, the cells were grown in liquid Superbroth supplemented with MVA and glucose at 37 °C to early stationary phase. The harvested cells were suspended in a solution containing 50 mM Tris HCl pH 7.8, 200 µg/ml lysozyme, and protease inhibitor cocktail (Nacalai Tesque, Japan), incubated for 5 min at 37 °C and lysed by sonication. Following centrifugation at 18,800 × *g* for 20 min at 4 °C, the lysates were subjected to native PAGE to separate holo- and apo-Fdx, and the proteins were determined by western blotting using specific antibody. The protein levels of SufB, SufC and SufD were determined by SDS-PAGE followed by Western blotting using specific antibodies. Detection was with ECL prime kit (GE Healthcare).

For pull-down assays between SufE and SufB, *E. coli* strain YT2512 (Δ*sufABCDSE*) was sequentially transformed with the plasmids pRK-*sufCDSE-*His and pBBR-*sufAB* (carrying mutations), and cultivated in LB medium at 37 °C to late log phase (OD_600_ = 0.2–0.3). The cells were harvested by centrifugation, suspended in a solution containing 50 mM Tris-HCl pH 7.8, 300 mM NaCl, 10 mM DTT, 20 mM imidazole, and protease inhibitor cocktail (Nacalai Tesque, Japan), and disrupted by lysozyme treatment and sonication. SufE-His protein was purified using Ni-affinity resin (COSMOGEL His-Accept, Nacalai Tesque, Japan) according to the manufacturer’s protocol. Aliquots of the eluate were subjected to Western blot analysis using specific antibodies against SufB and SufE.

### Protein expression and purification

The variant SufBCD complexes were expressed in the YT2512 (Δ*sufABCDSE*) cells from the pGSO164-derived plasmid carrying point mutations, and purified as described previously^25, 35^ with three steps of chromatography using Phenyl FF (low sub), Q XL, and Sephacryl S-200 columns (GE Healthcare). The SufS protein was also purified from the extract^28^. SufE was expressed (without His-tag sequence) from the pET21a-*sufE* plasmid in the HMS174(DE3) cells, and purified with two steps of chromatography using Phenyl FF (low sub) and Q XL columns (GE Healthcare). Protein concentration was determined by the dye binding method with Protein Assay CBB Solution (Nacalai Tesque, Japan) using bovine serum albumin as the standard protein.

### Cysteine desulfurase activity and sulfur-transfer assay

Cysteine desulfurase activity was determined by a previously reported method^25^. Briefly, reactions were carried out at 30 °C for 10 min in 25 mM Tris-HCl pH 7.8, 150 mM NaCl, 2 mM L-cysteine, 2 mM DTT, using 0.5 µM SufS, 2.0 µM SufE, and varying concentrations of the SufBCD complex. The liberated S^2−^ was converted to methylene blue and colorimetrically determined.

For the sulfur transfer assay from SufSE to SufBCD, DTT contained in the protein samples was removed before the reaction by ultrafiltration using Centri-Spin 10 (Nacalai Tesque, Japan). The reaction contained 50 mM Tris-HCl pH 7.8, 150 mM NaCl, 2 mM L-cysteine, 0.5 µM SufS, 0.5 µM SufE, and 10 µM SufBCD in a total volume of 125 μl. After incubation at 30 °C for 3 h, the SufBCD complex was separated from the other components by ultrafiltration with 100 kDa cutoff Amicon Ultra membrane (Millipore). The sample volume was adjusted to 125 μl and the SufBCD complex was determined using a molar absorption coefficient at 280 nm of 130 mM^−1^ cm^–1^. The S^0^ bound to the SufBCD complex was reduced to S^2−^ with 2 mM DTT, and then converted to methylene blue by the addition of 12.5 μl of 20 mM *N,N*-dimethyl-*p*-phenylenediamine in 7.2 M HCl and 12.5 μl of 30 mM FeCl_3_ in 1.2 M HCl. After incubation for 20 min, precipitated protein was removed by centrifugation at 18,800 × *g* for 1 min, and methylene blue was determined at 670 nm.

### Data availability statement

The datasets generated during the current study are available from the corresponding author on reasonable request.

## Electronic supplementary material

Supplementary Information
