# Supplementary material for: Mapping the key residues of SufB and SufD essential for biosynthesis of iron-sulfur clusters
Source: Sci Rep. 2017 Aug 24;7:9387. doi: 10.1038/s41598-017-09846-2 (PMC5571166; doi:10.1038/s41598-017-09846-2)
Supplement: Supplementary file 1 — Supplementary Information [file 41598_2017_9846_MOESM1_ESM.pdf]

Supplementary Information

Mapping the key residues of SufB and SufD essential for biosynthesis of iron-sulfur clusters.

Eiki Yuda[1], Naoyuki Tanaka[1,†], Takashi Fujishiro[1], Nao Yokoyama[1], Kei Hirabayashi[2,‡], Keiichi Fukuyama[3], Kei Wada[2], and Yasuhiro Takahashi[1*]

[1]*Department of Biochemistry and Molecular Biology, Graduate School of Science and Engineering, Saitama University, 255 Shimo-Okubo, Sakura-ku, Saitama, 338-8570, Japan*
[2]*Department of Medical Sciences, University of Miyazaki, Miyazaki 889-1692, Japan*
[3]*Department of Applied Chemistry, Graduate School of Engineering, Osaka University, Osaka 565-0871, Japan*
[†]*Present address: Innovation Medical Research Institute, University of Tsukuba, Ibaraki 305-8577, Japan*
[‡]*Present address: Department of Applied Biological Chemistry, Graduate School of Agricultural and Life Sciences, The University of Tokyo, Tokyo 113-8657, Japan*
* Corresponding author

Table S1. *E. coli* strains and plasmids used in this study. p2
Table S2. List of primers used in this study. p3
Table S3. Effect of site-directed mutations on the complementation of growth phenotype. p9
Table S4. Effect of suppressor mutations in *sufB* on the growth rate. p11
Table S5. Residues surrounding the tunnel inside the β-helix of SufB. p12
Figure S1. Temperature-sensitive growth phenotype elicited by amino acid substitutions in *sufB*. p13
Figure S2. Effect of point mutations in *sufD*. p14
Figure S3. Purification and characterization of the variant forms of the SufBCD complex. p15
Figure S4. Conservation of amino acid residues in SufB and SufD. p16

Table S1. *E. coli* strains and plasmids used in this study.

**Strain/plasmid**	**Description**	**Reference/source**
Strains		
MG1655	Wild type	Laboratory strain
UT109	MG1655 Δ(*iscUA-hscBA*)::Km^r Δ(*sufABCDSE*)::Gm^r	1
YT2512	MG1655 Δ(*sufABCDSE*)::Gm^r	2
HMS174(DE3)	F^-, *recA1*, *hsdR*(r_{K12}^- m_{K12}^+) (Rif^R) (DE3)	Novagen
Plasmids		
pUMV22 Sp^r	Sp^r; pUC19 derivative carrying three gene for MVA kinase, PMVA kinase and DPMVA decarboxylase.	3
pRKNMC	Tc^r; IncP-1 replicon, low-copy-number vector	4
pRK-SUF017	pRKNMC derivative carrying *E. coli sufABCDSE-ynhG*	2
pRK-*sufCDSE*	pRKNMC derivative carrying *E. coli sufCDSE*	This study
pRK-*sufCDSE*-His	pRK-*sufCDSE* carrying *sufE* fused with $(His)_6$-tag sequence	This study
pRK-*sufABC-SE*	pRKNMC derivative carrying *E. coli sufABC-SE*	5
pBBR1MCS-4	Ap^r; pBBR replicon, low-copy-number vector	6
pBBR-*sufAB*	pBBR1MCS-4 derivative carrying *E. coli sufAB*	This study
pBBR-*sufD*	pBBR1MCS-4 derivative carrying *E. coli sufD*	5
pGSO164	Ap^r; pBAD/*Myc*-His C derivative carrying *E. coli sufABCDSE*	7
pET21a-*sufE*	Ap^r; pET21a derivative carrying *E. coli sufE*	This study

References

1. Tokumoto, U., Kitamura, S., Fukuyama, K. & Takahashi, Y. Interchangeability and distinct properties of bacterial Fe-S cluster assembly systems: Functional replacement of the *isc* and *suf* operons in *Escherichia coli* with the *nifSU*-like operon from *Helicobacter pylori*. *J. Biochem.* **136,** 199–209 (2004).
2. Takahashi, Y. & Tokumoto, U. A third bacterial system for the assembly of iron-sulfur clusters with homologs in Archaea and plastids. *J. Biol. Chem.* **277,** 28380–28383 (2002).
3. Tanaka, N. *et al.* Novel features of the ISC machinery revealed by characterization of *Escherichia coli* mutants that survive without iron-sulfur clusters. *Mol. Microbiol.* **99,** 835–848 (2015).
4. Nakamura, M., Saeki, K. & Takahashi, Y. Hyperproduction of recombinant ferredoxins in *Escherichia coli* by coexpression of the ORF1-ORF2-*iscS-iscU-iscA-hscB-hscA-fdx*-ORF3 gene cluster. *J. Biochem.* **126,** 10–18 (1999).
5. Wada, K. *et al.* Molecular dynamism of Fe-S cluster biosynthesis implicated by the structure of the $SufC_2$-$SufD_2$ complex. *J. Mol. Biol.* **387,** 245–258 (2009).
6. Kovach, M. E. *et al.* Four new derivatives of the broad-host-range cloning vector pBBR1MCS, carrying different antibiotic-resistance cassettes. *Gene* **166,** 175–176 (1995).
7. Outten, F. W., Wood, M. J., Muñoz, F. M. & Storz, G. The SufE protein and the SufBCD complex enhance SufS cysteine desulfurase activity as part of a sulfur transfer pathway for Fe-S cluster assembly in *Escherichia coli*. *J. Biol. Chem.* **278,** 45713–45719 (2003).

Table S2. List of primers used in this study

Primer	Sequence
M13Rev	5’-CAGGAAACAGCTATGACC-3’
SufB-RSc3	5’-CTCCAGAGCTCCACTTAACATGTTTATTCCTTATCCGAC-3’
SufC-FSc2	5’-GCTCTAGAGCTCTGAACACAGCGTCGGATAAG-3’
SufER-Nh	5’-CCGGGCTAGCCAACCGGATGAAAGCTGT-3’
pRK-His6-F	5’-CATCATCATCATCATCATTAGCTAGCTAATTCACTGGCCGTC-3’
SufE-Cter-R	5’-GCTAAGTGCAGCGGCTTTGG-3’
EcSufE-F	5’-CATATGGCTTTATTGCCGGATAAAG-3’
EcSufE-R	5’-GGATCCTCGAGCTCAGCTAAGTGCAGCGGCTTTG-3’
SufB_E52A_F	5’-GGCGAAGCGTAATGCGCCGGAGTGGATG-3’
SufB_E52A_R	5’-CATCCACTCCGGCGCATTACGCTTCGCC-3’
SufB_M56A_F	5’-GCGCTGGAGTTTCGTCTAAACGCC-3’
SufB_M56-R	5’-CCACTCCGGCTCATTACGC-3’
SufB_R60A_F	5’-GAGTGGATGCTGGAGTTTGCTCTAAACGCCTATCGCGC-3’
SufB_R60A_R	5’-GCGCGATAGGCGTTTAGAGCAAACTCCAGCATCCACTC-3’
SufB_C96A_F	5’-GCCGGTAATTGTGACGACACTTGCGCGTC-3’
SufB_C96_R	5’-CGATGGTGCTGAGTAGTAGCTGTAATC-3’
SufB_C99A_F	5’-CACCATCGTGCGGTAATGCTGACGACACTTGCGCG-3’
SufB_C99A_R	5’-CGCGCAAGTGTCGTCAGCATTACCGCACGATGGTG-3’
SufB_C103A_F	5’-GCCGCGTCTGAACCTGGCGCGGTGC-3’
SufB_C103_R	5’-AGTGTCGTCACAATTACCGCACGATG-3’
SufB_C167A_F	5’-CGGAGCAGGGAATTATTTTCGCTTCCTTTGGTGAGGCGA-3’
SufB_C167A_R	5’-TCGCCTCACCAAAGGAAGCGAAAATAATTCCCTGCTCCG-3’
SufB_H176A_F	5’-GAGGCGATCCACGATGCCCCGGAACTGGTGC-3’
SufB_H176A_R	5’-GCACCAGTTCCGGGGCATCGTGGATCGCCTC-3’
SufB_D192A_F	5’-GCCAACTTCTTTGCCGCGCTTAATGCG-3’
SufB_D192_R	5’-ATTCCCCGGCACCACGGTGCCGAGATATTTAC-3’
SufB_C217A_F	5’-CCTAAAGGCGTGCGCGCCCCGATGGAACTTTCC-3’
SufB_C217A_R	5’-GGAAAGTTCCATCGGGGCGCGCACGCCTTTAGG-3’
SufB_Y224A_F	5’-GCTTTTCGCATTAACGCAGAAAAAACCGGGCAGTTTG-3’
SufB_Y224_R	5’-GGTGGAAAGTTCCATCGGGCAGCGC-3’
SufB_R226A_F	5’-GCCATTAACGCAGAAAAAACCGGGCAGTTTGAGC-3’
SufB-R226X_F	5’-NNKATTAACGCAGAAAAAACCGGGCAG-3’
SufB_R226_R	5’-AAAATAGGTGGAAAGTTCCATCGGGC-3’
SufB_N228A_F	5’-GCCGCAGAAAAAACCGGGCAGTTTGAGCG-3’

Table S2. Cont.

Primer	Sequence
SufB_N228X_F	5’-NNKGCAGAAAAAACCGGGCAGTTTGA-3’
SufB_N228_R	5’-AATGCGAAAATAGGTGGAAAGTTCCATCG-3’
SufB_E230A_F	5’-GCAAAAACCGGGCAGTTTGAGCGCAC-3’
SufB_E230_R	5’-TGCGTTAATGCGAAAATAGGTGGAAAGTTC-3’
SufB_Q234A_F	5’-CGCAGAAAAAACCGGGGCGTTTGAGCGCACCATTCTG-3’
SufB_Q234A_R*	5’-CAGAATGGTGCGCTCAAACGCCCCGGTTTTTTCTGCG-3’
SufB_E236A_F	5’-CCGGGCAGTTTGCGCGCACCATTCTGG-3’
SufB_E236A_R	5’-CCAGAATGGTGCGCGCAAACTGCCCGG-3’
SufB_R237A_F	5’-GCCACCATTCTGGTGGCCGACGAAGACAG-3’
SufB_R237_R	5’-CTCAAACTGCCCGGTTTTTTCTGCG-3’
SufB_T238A_F	5’-GCCATTCTGGTGGCCGACGAAGACAG-3’
SufB_T238_R	5’-GCGCTCAAACTGCCCGGTTTTTTCTG-3’
SufB_D243A_F	5’-GCCGAAGACAGCTACGTCAGCTACATTG-3’
SufB_D243_R	5’-GGCCACCAGAATGGTGCGCTCAAAC-3’
SufB_E244A_F	5’-GCAGACAGCTACGTCAGCTACATTGAAGG-3’
SufB_E244_R	5’-GTCGGCCACCAGAATGGTGCGCTCAAAC-3’
SufB_Y250A_F	5’-GCCATTGAAGGCTGTTCCGCTCCGGTGC-3’
SufB_Y250_R	5’-GCTGACGTAGCTGTCTTCGTCGGCC-3’
SufB_E252A_F	5’-GCAGGCTGTTCCGCTCCGGTGCGTG-3’
SufB_E252_R	5’-AATGTAGCTGACGTAGCTGTCTTCGTC-3’
SufB_C254A_F	5’-GTCAGCTACATTGAAGGCGCTTCCGCTCCGGTGC-3’
SufB_C254A_R	5’-GCACCGGAGCGGAAGCGCCTTCAATGTAGCTGAC-3’
SufB_C254X_F	5’-NNKTCCGCTCCGGTGCGTGA-3’
SufB_C254_R	5’-GCCTTCAATGTAGCTGACGTAG-3’
SufB_H265A_F	5’-GCGTGACAGCTATCAGTTAGCCGCGGCAGTGGTG-3’
SufB_H265A_R	5’-CACCACTGCCGCGGCTAACTGATAGCTGTCACGC-3’
SufB_E270A_F	5’-GCAGTCATCATCCATAAAAACGCCGAGGTG-3’
SufB_E270_R	5’-CACCACTGCCGCGTGTAACTGATAG-3’
SufB_Y281A_F	5’-GCTTCCACGGTACAAAACTGGTTTCCTGGCG-3’
SufB_Y281_R	5’-TTTCACCTCGGCGTTTTTATGGATGATGAC-3’
SufB_T283A_F	5’-GCGGTACAAAACTGGTTTCCTGGCGATAAC-3’
SufB_T283_R	5’-GGAATATTTCACCTCGGCGTTTTTATGGATG-3’
SufB_Q285A_F	5’-GCAAACTGGTTTCCTGGCGATAACAACACCGG-3’
SufB_Q285X_F	5’-NNKAACTGGTTTCCTGGCGATAACAAC-3’

Table S2. Cont.

**Primer**	**Sequence**
SufB_Q285_R	5’-TACCGTGGAATATTTCACCTCGGCG-3’
SufB_N286A_F	5’-GAAATATTCCACGGTACAAGCCTGGTTTCCTGGCGATAACAAC-3’
SufB_N286A_R	5’-GTTGTTATCGCCAGGAAACCAGGCTTGTACCGTGGAATATTTC-3’
SufB_W287A_F	5’-GCGTTTCCTGGCGATAACAACACCGGCGG-3’
SufB_W287X_F	5’-NNKTTTCCTGGCGATAACAACACCG-3’
SufB_W287_R	5’-GTTTTGTACCGTGGAATATTTCACCTCGG-3’
SufB_N299A_F	5’-GCCTTCGTCACCAAGCGTGCTTTGTGCGAA-3’
SufB_N299_R	5’-GAGAATACCGCCGGTGTTGTTATCG-3’
SufB_T302A_F	5’-GCCAAGCGTGCTTTGTGCGAAGGCG-3’
SufB_T302_R	5’-GACGAAGTTGAGAATACCGCCGGTG-3’
SufB_K303A_F	5’-GCGCGTGCTTTGTGCGAAGGCGAAAACAG-3’
SufB_K303X_F	5’-NNKCGTGCTTTGTGCGAAGGCGA-3’
SufB_K303_R	5’-GGTGACGAAGTTGAGAATACCGCCG-3’
SufB_R304A_F	5’-CTCAACTTCGTCACCAAGGCTGCTTTGTGCGAAGGCG-3’
SufB_R304A_R	5’-CGCCTTCGCACAAAGCAGCCTTGGTGACGAAGTTGAG-3’
SufB_C307A_F	5’-GTCACCAAGCGTGCTTTGGCCGAAGGCGAAAACAGC-3’
SufB_C307A_R	5’-GCTGTTTTCGCCTTCGGCCAAAGCACGCTTGGTGAC-3’
SufB_W316A_F	5’-GCGAAAACAGCAAAATGTCAGCGACGCAATCAGAAACCGGG-3’
SufB_W316A_R	5’-CCCGGTTTCTGATTGCGTCGCTGACATTTTGCTGTTTTCGC-3’
SufB_S323A_F	5’-GCAGCGATTACGTGGAAATATCCCAGCTG-3’
SufB_S323_R	5’-CCCGGTTTCTGATTGCGTCCATGAC-3’
SufB_T326A_F	5’-GCGTGGAAATATCCCAGCTGCATTTTGCG-3’
SufB_T326_R	5’-AATCGCTGACCCGGTTTCTGATTGC-3’
SufB_K328A_F	5’-GCATATCCCAGCTGCATTTTGCGCGGCGATAAC-3’
SufB_K328_R	5’-CCACGTAATCGCTGACCCGGTTTCTG-3’
SufB_Y329A_F	5’-GCGATTACGTGGAAAGCTCCCAGCTGCATTTTGCGCG-3’
SufB_Y329A_R	5’-CGCGCAAAATGCAGCTGGGAGCTTTCCACGTAATCGC-3’
SufB_C332A_F	5’-CGTGGAAATATCCCAGCGCCATTTTGCGCGGCGATAAC-3’
SufB_C332A_R	5’-GTTATCGCCGCGCAAAATGGCGCTGGGATATTTCCACG-3’
SufB_S345A_F	5’-GCAGTGGCGCTGACCAGCGGTCATC-3’
SufB_S345_R	5’-GTAAAACTCACCAATGGAGTTATCGCCG-3’
SufB_Q353A_F	5’-GCGCAAGCGGATACCGGCACCAAGATGATC-3’
SufB_Q353_R	5’-ATGACCGCTGGTCAGCGCCACTGAG-3’
SufB_D356A_F	5’-GTCATCAGCAAGCGGCTACCGGCACCAAG-3’

Table S2. Cont.

Primer	Sequence
SufB_D356A_R	5’-CTTGGTGCCGGTAGCCGCTTGCTGATGAC-3’
SufB_K360A_F	5’-GCGATGATCCACATCGGTAAAAACACCAAATCGACC-3’
SufB_K360_R	5’-GGTGCCGGTATCCGCTTGCTGATGAC-3’
SufB_M361A_F	5’-GCGATCCACATCGGTAAAAACACCAAAT-3’
SufB_M361_R	5’-CTTGGTGCCGGTATCCGCTT-3’
SufB_H363A_F	5’-CCGGCACCAAGATGATCGCCATCGGTAAAAACACCAAATC-3’
SufB_H363A_R	5’-GATTTGGTGTTTTTACCGATGGCGATCATCTTGGTGCCGG-3’
SufB_T368A_F	5’-GCCAAATCGACCATTATCTCGAAAGGGATCTC-3’
SufB_T368_R	5’-GTTTTTACCGATGTGGATCATCTTGGTGC-3’
SufB_S370A_F	5’-GCGACCATTATCTCGAAAGGGATCTCTGC-3’
SufB_S370_R	5’-TTTGGTGTTTTTACCGATGTGGATCATCTTG-3’
SufB_K375A_F	5’-GCAGGGATCTCTGCCGGACATAGTCAGAAC-3’
SufB_K375_R	5’-CGAGATAATGGTCGATTTGGTGTTTTTACC-3’
SufB_Y386A_F	5’-GCTCGCGGCTTAGTGAAAATCATGCCGACG-3’
SufB_Y386_R	5’-ACTGTTCTGACTATGTCCGGCAGAG-3’
SufB_R387A_F	5’-GCCGGCTTAGTGAAAATCATGCCGACGGC-3’
SufB_R387_R	5’-ATAACTGTTCTGACTATGTCCGGCAG-3’
SufB_C405A_F	5’-GCGCGCAATTTCACTCAGGCCGACTCAATGCTGATTGG-3’
SufB_C405A_R	5’-CCAATCAGCATTGAGTCGGCCTGAGTGAAATTGCGCGC-3’
SufB_C405X_F	5’-NNKGACTCAATGCTGATTGGCGCTAAT-3’
SufB_C405_R	5’-CTGAGTGAAATTGCGCGCATTG-3’
SufB_D406A_F	5’-GCCTCAATGCTGATTGGCGCTAATTGTGG-3’
SufB_D406_R	5’-GCACTGAGTGAAATTGCGCGCATTG-3’
SufB_C414A_F	5’-CAATGCTGATTGGCGCTAATGCTGGGGCGCATACCTTC-3’
SufB_C414A_R	5’-GAAGGTATGCGCCCCAGCATTAGCGCCAATCAGCATTG-3’
SufB_H417A_F	5’-CGCTAATTGTGGGGCGGCTACCTTCCCGTATGTTGAG-3’
SufB_H417A_R	5’-CTCAACATACGGGAAGGTAGCCGCCCCACAATTAGCG-3’
SufB_T418A_F	5’-GCCTTCCCGTATGTTGAGTGTCGTAACAATAG-3’
SufB_T418_R	5’-ATGCGCCCCACAATTAGCGCCAATC-3’
SufB_Y421A_F	5’-GCTGTTGAGTGTCGTAACAATAGTGCGCAACTGG-3’
SufB_Y421_R	5’-CGGGAAGGTATGCGCCCCACAATTAG-3’
SufB_N426A_F	5’-GCCAATAGTGCGCAACTGGAACACGAGGC-3’
SufB_N426_R	5’-ACGACACTCAACATACGGGAAGGTATG-3’
SufB_E432A_F	5’-GCACACGAGGCAACGACATCACGTATTG-3’

Table S2. Cont.

Primer	Sequence
SufB_E432A/H433A_F	5'-GCAGCCGAGGCAACGACATCAC-3'
SufB_E432_R	5'-CAGTTGCGCACTATTGTTACGACACTC-3'
SufB_H433A_F	5'-CAATAGTGCGCAACTGGAAGCCGAGGCAACGACATCAC-3'
SufB_H433A_R	5'-GTGATGTCGTTGCCTCGGCTTCCAGTTGCGCACTATTG-3'
SufB_E434A_F	5'-GCGGCAACGACATCACGTATTGGTGAAG-3'
SufB_E434X_F	5'-NNKGCAACGACATCACGTATTGGTGAA-3'
SufB_E434_R	5'-GTGTTCCAGTTGCGCACTATTGTTAC-3'
SufB_S438A_F	5'-GCACGTATTGGTGAAGATCAACTGTTTTACTGCC-3'
SufB_S438_R	5'-TGTCGTTGCCTCGTGTTCCAGTTGC-3'
SufB_Y447A_F	5'-GCCTGCCTGCAACGCGGGATCAGCGAAG-3'
SufB_Y447_R	5'-AAACAGTTGATCTTCACCAATACGTGATGTC-3'
SufB_C448A_F	5'-GGTGAAGATCAACTGTTTTACGCCCTGCAACGCGGGATC-3'
SufB_C448A_R	5'-GATCCCGCGTTGCAGGGCGTAAAACAGTTGATCTTCACC-3'
SufB_R451A_F	5'-GCCGGGATCAGCGAAGAAGACGCCATCTC-3'
SufB_R451_R	5'-TTGCAGGCAGTAAAACAGTTGATCTTCAC-3'
SufB_C467A_F	5'-CGATGATTGTTAACGGTTTCGCCAAAGACGTGTTCTCGGAG-3'
SufB_C467A_R	5'-CTCCGAGAACACGTCTTTGGCGAAACCGTTAACAATCATCG-3'
SufB_E477A_F	5'-CGGAGCTGCCGTTGGCATTTGCCGTTGAAG-3'
SufB_E477A_R	5'-CTTCAACGGCAAATGCCAACGGCAGCTCCG-3'
SufD_D283A_F	5'-GCTACCCGTACCTGGCTGG-3'
SufD_D283_R	5'-ACACACCTCGTTTTTCACCGGC-3'
SufD_H290A_F	5'-GCCAATAAAGGTTTTTGTAACAGCCGAC-3'
SufD_H290_R	5'-TTCCAGCCAGGTACGGGTATC-3'
SufD_K302A_F	5'-GCAACTATCGTCAGCGACAAAGGC-3'
SufD_K302_R	5'-GTGCAACTGTCGGCTGTTACAAAAAC-3'
SufD_D344A_F	5'-GCTACGAAACCGCAGCTGGAAATC-3'
SufD_D344_R	5'-CACTTCCGCCAGTTTGCCC-3'
SufD_E350A_F	5'-GCAATCTATGCAGATGATGTGAAATGC-3'
SufD_E350_R	5'-CAGCTGCGGTTTCGTATCCAC-3'
SufD_D354A_F	5'-GCTGATGTGAAATGCAGCCACG-3'
SufD_D354_R	5'-TGCATAGATTTCCAGCTGCGG-3'
SufD_D355A_F	5'-GCTGTGAAATGCAGCCACGGC-3'
SufD_D355_R	5'-ATCTGCATAGATTTCCAGCTGCG-3'
SufD_C358A_F	5'-GCCAGCCACGGCGCGACG-3'

Table S2. Cont.

Primer	Sequence
SufD_C358_R	5'-TTTCACATCATCTGCATAGATTTCCAG-3'
SufD_C358S_F	5'-CAGATGATGTGAAATCGAGCCACGGCGCGACGG-3'
SufD_C358S_R	5'-CCGTCGCGCCGTGGCTCGATTTCACATCATCTG-3'

The underlines indicate the altered codons for site-directed mutagenesis. The double underlined bases indicate restriction sites.

Table S3. Effect of site-directed mutations on the complementation of growth phenotype

Substitution	Complementation	Substitution	Complementation
$SufB^{E52A}$	Partial	$SufB^{R304A}$	Yes
$SufB^{M56A}$	Yes	$SufB^{C307A}$	Yes
$SufB^{R60A}$	Partial	$SufB^{W316A}$	Partial
$SufB^{C96A}$	Yes	$SufB^{S323A}$	Yes
$SufB^{C99A}$	Yes	$SufB^{T326A}$	Partial
$SufB^{C103A}$	Yes	$SufB^{K328A}$	Partial
$SufB^{C167A}$	Yes	$SufB^{Y329A}$	Yes
$SufB^{H176A}$	Yes	$SufB^{C332A}$	Yes
$SufB^{D192A}$	Partial	$SufB^{S345A}$	Yes
$SufB^{C217A}$	Yes	$SufB^{Q353A}$	Partial
$SufB^{Y224A}$	Partial (TS)	$SufB^{D356A}$	Partial
$SufB^{R226A}$	No	$SufB^{K360A}$	Yes
$SufB^{N228A}$	No	$SufB^{M361A}$	Yes
$SufB^{E230A}$	Yes	$SufB^{H363A}$	Yes
$SufB^{Q234A}$	Partial (TS)	$SufB^{T368A}$	Yes
$SufB^{E236A}$	Partial (TS)	$SufB^{S370A}$	Yes
$SufB^{R237A}$	Partial	$SufB^{K375A}$	Yes
$SufB^{T238A}$	Yes	$SufB^{Y386A}$	Yes
$SufB^{D243A}$	Partial	$SufB^{R387A}$	Partial
$SufB^{E244A}$	Yes	$SufB^{C405A}$	No
$SufB^{Y250A}$	Yes	$SufB^{D406A}$	Yes
$SufB^{E252A}$	Partial (TS)	$SufB^{C414A}$	Yes
$SufB^{C254A}$	No	$SufB^{H417A}$	Yes
$SufB^{H265A}$	Partial	$SufB^{T418A}$	Yes
$SufB^{E270A}$	Partial	$SufB^{Y421A}$	Yes
$SufB^{Y281A}$	Partial	$SufB^{N426A}$	Yes
$SufB^{T283A}$	Partial	$SufB^{E432A}$	Yes
$SufB^{Q285A}$	No	$SufB^{H433A}$	Yes
$SufB^{N286A}$	Partial	$SufB^{E434A}$	No
$SufB^{W287A}$	No	$SufB^{S438A}$	Yes
$SufB^{N299A}$	Partial	$SufB^{Y447A}$	Yes
$SufB^{T302A}$	Yes	$SufB^{C448A}$	Yes
$SufB^{K303A}$	No	$SufB^{R451A}$	Yes

Table S3. Cont.

Substitution	Complementation
SufB^{C467A}	Yes
SufB^{E477A}	Yes
SufB$^{\Delta96\text{-}103}$	Yes
SufD^{D283A}	Yes
SufD^{H290A}	Yes
SufD^{K302A}	Yes
SufD^{D344A}	Yes
SufD^{E350A}	Yes
SufD^{D354A}	Yes
SufD^{D355A}	Yes
SufD^{C358A}	Yes
SufD^{H360A}	No
SufD^{H360S}	No
SufD^{H360C}	No
SufB^{C307A}/SufB^{C332A}	Yes
SufB^{D406A}/SufB^{E432A}	Yes
SufB^{D406A}/SufB^{H433A}	Yes
SufB^{E432A}/SufB^{H433A}	Partial
SufB^{D406A}/SufD^{C358A}	Yes
SufB^{D406A}/SufD^{C358S}	Yes
SufB^{E432A}/SufD^{C358A}	Yes

Substitution	Complementation
SufB^{E432A}/SufD^{C358S}	Yes
SufB^{H433A}/SufD^{C358A}	Yes
SufB^{H433A}/SufD^{C358S}	Yes
SufB^{D406A}/SufB^{E432A} /SufB^{H433A}	Partial
SufB^{D406A}/SufB^{E432A} /SufD^{C358A}	Yes
SufB^{D406A}/SufB^{E432A} /SufD^{C358S}	Yes
SufB^{D406A}/SufB^{H433A} /SufD^{C358A}	Yes
SufB^{D406A}/SufB^{H433A} /SufD^{C358S}	Yes
SufB^{E432A}/SufB^{H433A} /SufD^{C358A}	Partial
SufB^{E432A}/SufB^{H433A} /SufD^{C358S}	Partial
SufB^{D406A}/SufB^{E432A} /SufB^{H433A}/SufD^{C358A}	Partial
SufB^{D406A}/SufB^{E432A} /SufB^{H433A}/SufD^{C358S}	Partial

Table S4. Effect of suppressor mutations in *sufB* on the growth rate

Substitution	Doubling time (min)
R226V	119 ± 4.47
R226I	81.9 ± 3.53
R226L	96.3 ± 4.74
N228C	115 ± 3.38
N228H	84.0 ± 22.1
N228Q	105 ± 6.48
Q285G	53.5 ± 4.05
Q285N	74.0 ± 14.0
Q285R	239 ± 6.67
W287Y	140 ± 22.4
W287M	81.9 ± 9.46
W287F	43.3 ± 6.95
K303E	101 ± 2.53
K303R	49.8 ± 6.95
E434D	53.8 ± 3.09
Wild type SufB	34.0 ± 3.08

Table S5. Residues surrounding the tunnel inside the β-helix of SufB

SufB residue	Main/side chain	Conservation (%)	Complementation[a]
E236	Side	99.2	Partial
E252	Side	99.6	Partial
C254	Side	100	No
H265	Side	100	Partial
A267	Side	82.7	Not done
T283	Side	98.3	Partial
Q285	Side	97.5	No
K303	Side	95.4	No
Q318	Side	54.9	Not done
S319	Main	12.2	Not done
E320	Side	55.3	Not done
T326	Side	95.8	Partial
K328	Side	91.6	Partial
S345	Side	91.6	Yes
A347	Side	93.3	Not done
A355	Side	46.0	Not done
T357	Side	67.9	Not done
S374	Side	71.7	Not done
G376	Main/side	53.6	Not done
N384	Side	54.9	Not done
S385	Main	27.0	Not done
Y386	Side	94.5	Yes
C405	Side	100	No
D406	Main	96.6	Yes
S407	Side	58.7	Not done
T418	Side	98.3	Yes

[a]Effect of Ala-substitution on the growth phenotype

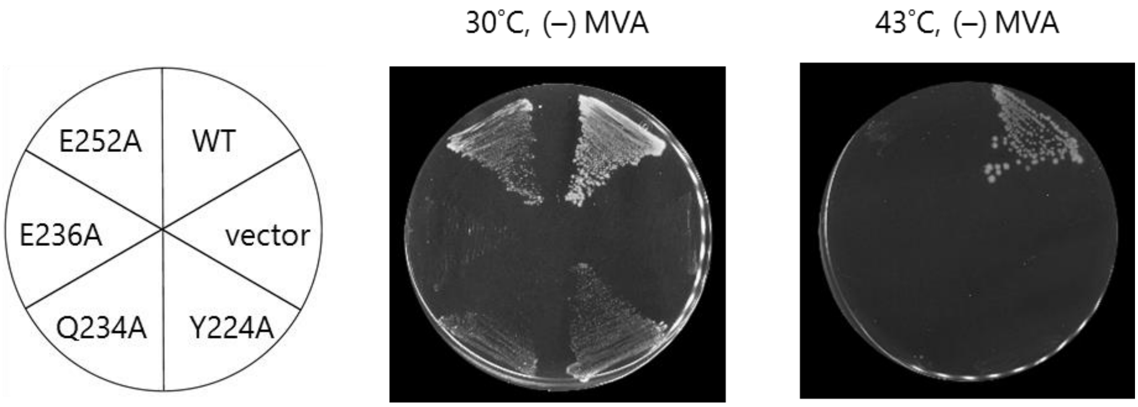

Figure S1. Temperature-sensitive growth phenotype elicited by amino acid substitutions in SufB. The UT109 cells harboring pUMV22 Sp^r were sequentially transformed with the pRK-*sufCDSE* plasmid and the pBBR-*sufAB* plasmid carrying a point mutation in *sufB* ($SufB^{Y224A}$, $SufB^{Q234A}$, $SufB^{E236A}$, or $SufB^{E252A}$). The cells were grown on LB plates (without MVA) at 30˚C or 43˚C for 48 h.

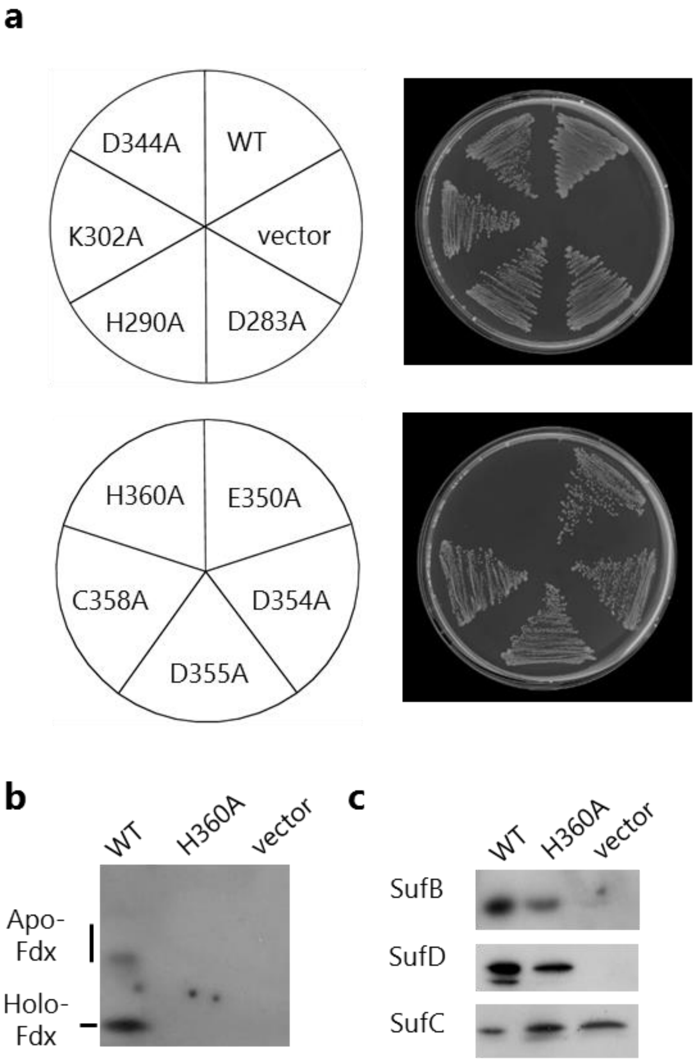

Figure S2. Effect of point mutations in *sufD*. The pRK-*sufABC-SE* plasmid and the pBBR-*sufD* plasmid carrying a point mutation in *sufD* were introduced into UT109 cells harboring pUMV22 Sp^r. (a) Growth of cells carrying the mutations. The cells were cultivated on LB plates (without MVA) at 37˚C for 24 h. (b) [2Fe-2S] holo-Fdx in the cells and (c) protein levels of SufB, SufD and SufC were determined as described in the legend to Figure 1.

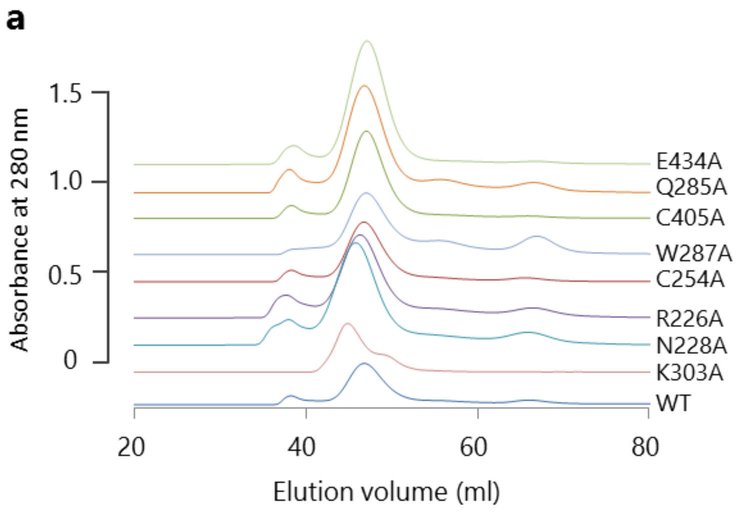

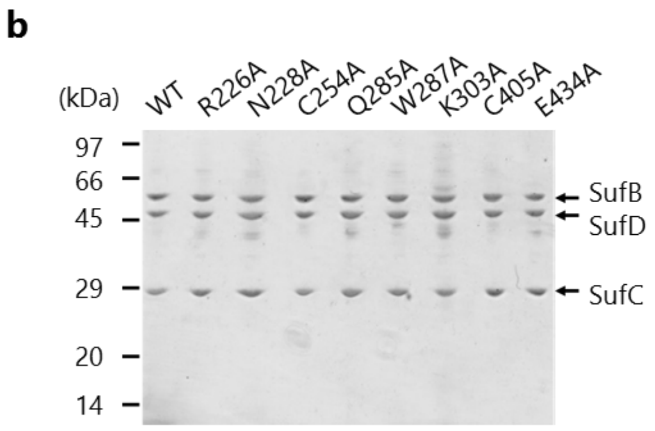

Figure S3. Purification and characterization of the variant forms of the SufBCD complex. (a) Comparison of the elution profiles from the Sephacryl S-200 gel-filtration column. (b) SDS-PAGE analysis of the variant forms of the SufBCD complex on a 12.5% gel.

a

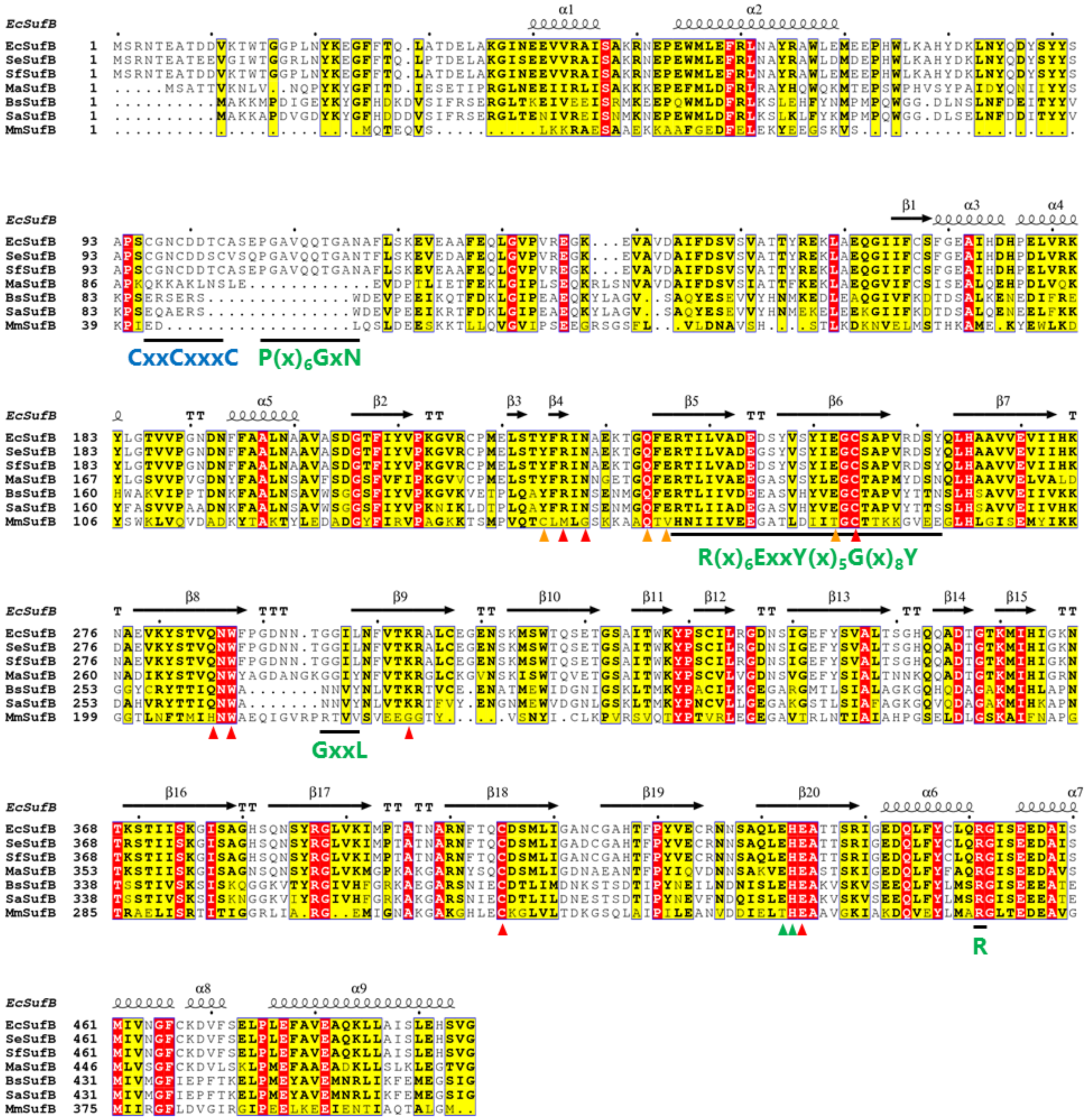

b

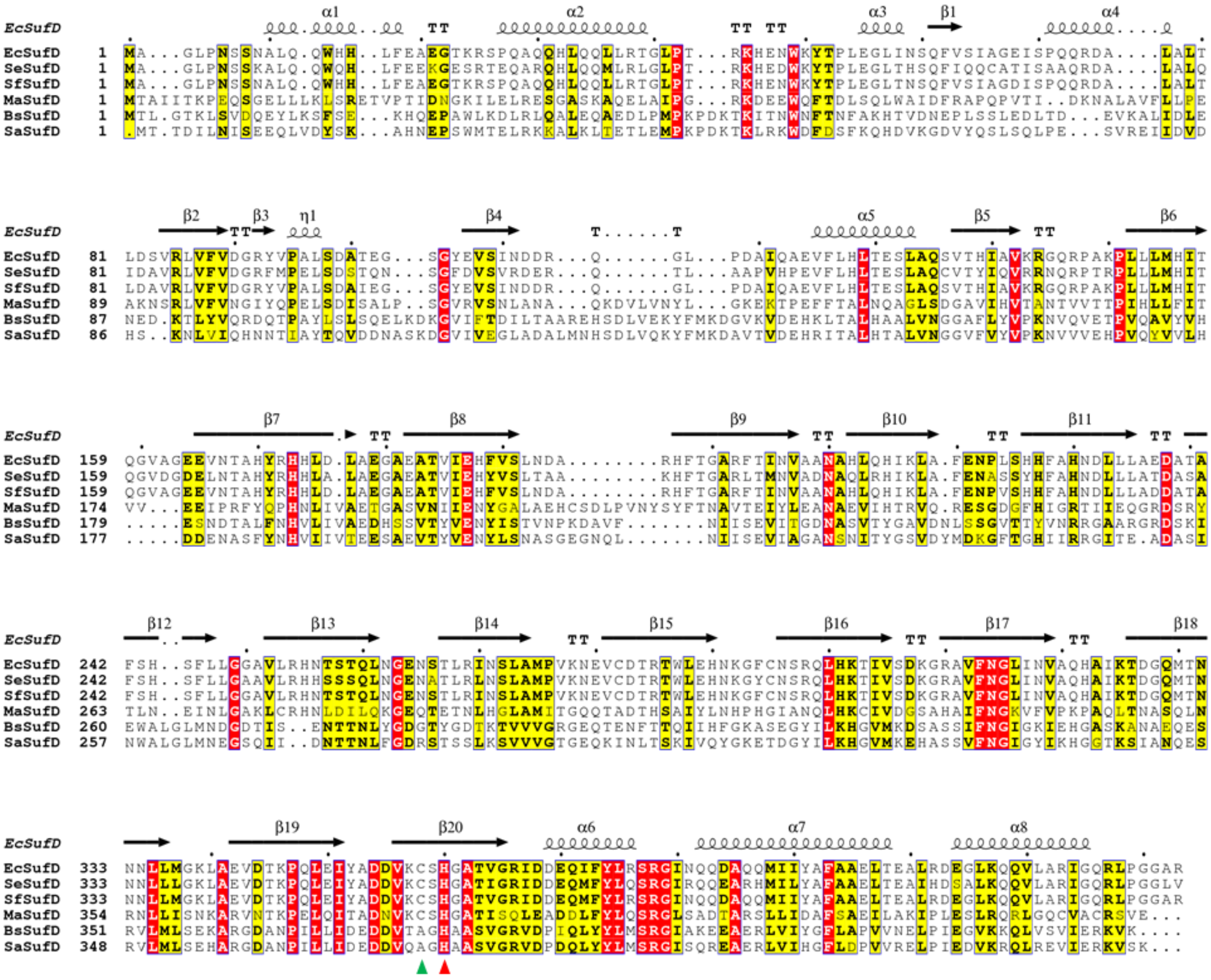

Figure S4. Conservation of amino acid residues in SufB and SufD. Multi-sequence alignments are provided for SufB (a) and for SufD (b). The functionally critical residues of *E. coli* SufB and SufD identified in this study are indicated by red triangles. The temperature-sensitive mutations are indicated in yellow and functionally redundant residues in green. The putative Fe-S cluster binding motif (CxxCxxxC) and the putative $FADH_2$ binding motif are indicated in blue and green characters, respectively. Abbreviations: Ec, *Escherichia coli*; Se, *Salmonella enterica*; Sf, *Shigella flexneri*; Ma, *Microcystis aeruginosa*; Bs, *Bacillus subtilis*; Sa, *Staphylococcus aureus*; Mm, *Methanosarcina mazei*.
